# A comprehensive mechanistic multicellular model of the human immune system spanning 11 diseases

**DOI:** 10.3389/fimmu.2026.1732556

**Published:** 2026-05-11

**Authors:** Robert Moore, Lauren Mayo, Dennis Startsev, Bhanwar Lal Puniya, Kashish Poore, Resa Helikar, Tomáš Helikar

**Affiliations:** Department of Biochemistry, University of Nebraska-Lincoln, Lincoln, NE, United States

**Keywords:** computational modeling, digital twin, immune system, logical modeling, systems immunology

## Abstract

**Introduction:**

The immune system is a dynamic, complex network of interacting cells, molecules, and signals central to health. Immune digital twins - virtual representations of the immune system that evolve in tandem with their biological counterparts - offer a path toward predictive, patient-specific simulations, but their realization requires mechanistic frameworks that generalize across multiple cell types and disease contexts.

**Methods:**

We constructed a logic-based mechanistic model of the human immune system built exclusively on human experimental data extracted from 449 publications. The model integrates 51 innate (e.g., NK cells, macrophages) and 37 adaptive (e.g., Th1/2/17, B cells) immune cell types and subtypes, together with 37 secretory factors and 1,450 regulatory interactions across 11 disease conditions, including nine pathogens, type 1 diabetes, and lung transplantation. Model predictions were validated against independent in vitro, ex vivo, and clinical observations not used during construction.

**Results:**

Across 38 validation experiments, agreement with published literature ranged from 75% to 90% across pathogens. The model reproduced pathogen-specific cytokine signatures for nine infections, captured synergistic and antagonistic effects in four coinfection scenarios (MTB–HIV, MTB–Helminth, SARS-CoV-2–EBV, and *Plasmodium falciparum*-Helminth), and resolved competing rejection and tolerance signals in lung transplantation challenged by CMV, EBV, and SARS-CoV-2.

**Discussion:**

This reference model unifies multiple immune contexts within a single simulatable model and generates a structured catalog of falsifiable predictions for experimental follow-up. The model is openly available on Cell Collective and as an SBML file, representing a step toward a clinically integrated immune digital twin for patient-specific applications.

## Introduction

The immune system is a multifaceted network of interacting cells, cytokines, and signaling pathways that collectively protect the body against pathogens, maintain tissue homeostasis, and facilitate wound healing. It comprises two closely interconnected branches, innate and adaptive immunity, linked by both direct cell-cell interactions (e.g., antigen-presenting cells engaging T cells via major histocompatibility complex molecules) and secretory factors (e.g., cytokines, immunoglobulins) that coordinate robust yet regulated responses (1). Maintaining the appropriate balance between inflammation and tolerance is essential; its dysfunction can drive pathologies such as type 1 diabetes (T1D), where autoreactive T cells attack pancreatic beta cells (2), or complications in transplantation, where immunosuppressive treatments to prevent graft rejection increase vulnerability to infections and chronic organ damage (3). The COVID-19 pandemic further underscores how viral evasion and dysregulated cytokine production can result in severe outcomes (4).

Harnessing the immune system for disease management is increasingly recognized as an effective therapeutic approach. Immunotherapy encompasses a variety of approaches, including monoclonal antibodies, immune cell-based therapy, checkpoint inhibitors, cytokine therapy, and vaccines (5). In the case of T1D, several clinical trials focus on restoring immunosuppressive cells, either by adoptively transferring regulatory T cells (Treg), by stimulating differentiation toward tolerogenic dendritic cells, or by administering the cytokine IL-2 to enhance the frequency of Treg (6). While the most common treatments for infections are vaccines and antibiotics, there are several U.S Food and Drug Administration (FDA)-approved blocking antibodies against several pathogens, including Ebola, and respiratory syncytial virus (7). Yet, the non-linear, multiscale nature of immune responses makes it difficult to predict their behavior, particularly under novel or combinatorial perturbations. High-throughput experimental approaches continue to generate large datasets, but these snapshots often fail to capture the full complexity of immune dynamics over time, across different diseases, and across patient populations. In digital medicine, medical digital twins—computational representations of complex biological processes—have emerged as an innovative approach for simulating patient-specific immune dynamics in silico (8, 9).

An effective immune digital twin must be grounded in a mechanistic framework that not only captures the dynamic cross-talk between innate and adaptive immunity, but also generalizes across disease contexts and perturbations. To enable predictive and personalized simulations, the model must align with experimental data for validation and efficiently integrate patient-specific variables. While computational models of the immune system have used logical, flux-based, or kinetic approaches, many focus on a limited set of cell types or a single disease. For instance, mechanistic models of CD4+ T cells predicted novel phenotypes and responses to different cytokine doses, including signaling pathways regulating differentiation (10). Other examples include multiscale models predicting the mucosal immune response to *Helicobacterpylori* infection (11) and the CD4+ T cell response to influenza infection (12). Logical models offer an approach to modeling large-scale biological systems due to their scalability and the ability to work without precise kinetic parameters, which are often difficult to obtain (13). By relying on qualitative or semi-quantitative regulatory rules rather than detailed biochemical rates, these models can effectively capture complex biological behaviors, including signaling cascades, gene regulatory networks, and immune responses. Additionally, logical models have been applied to various translational model-capturing networks from a few to a hundred components (14).

Here, we present a large-scale logic-based model of the human immune system, derived from human experimental data, that spans multiple disease contexts, including viral infections, T1D, and lung transplantation. This model captures direct and indirect communication pathways (e.g., cell-cell interactions and secretory factors) between innate and adaptive immunity. To demonstrate robustness, we validated predictions against independent experimental observations not used in model construction and then examined diverse scenarios, such as multiplex cytokine profiles for nine pathogens, coinfections, and transplantation challenged by three clinically relevant viruses. The model is available for direct *in silico* experimentation on the online modeling platform, Cell Collective (15), and in this manuscript as an SBML (16) file.

## Results

### Integrative model of the immune system

**Figure 1:**
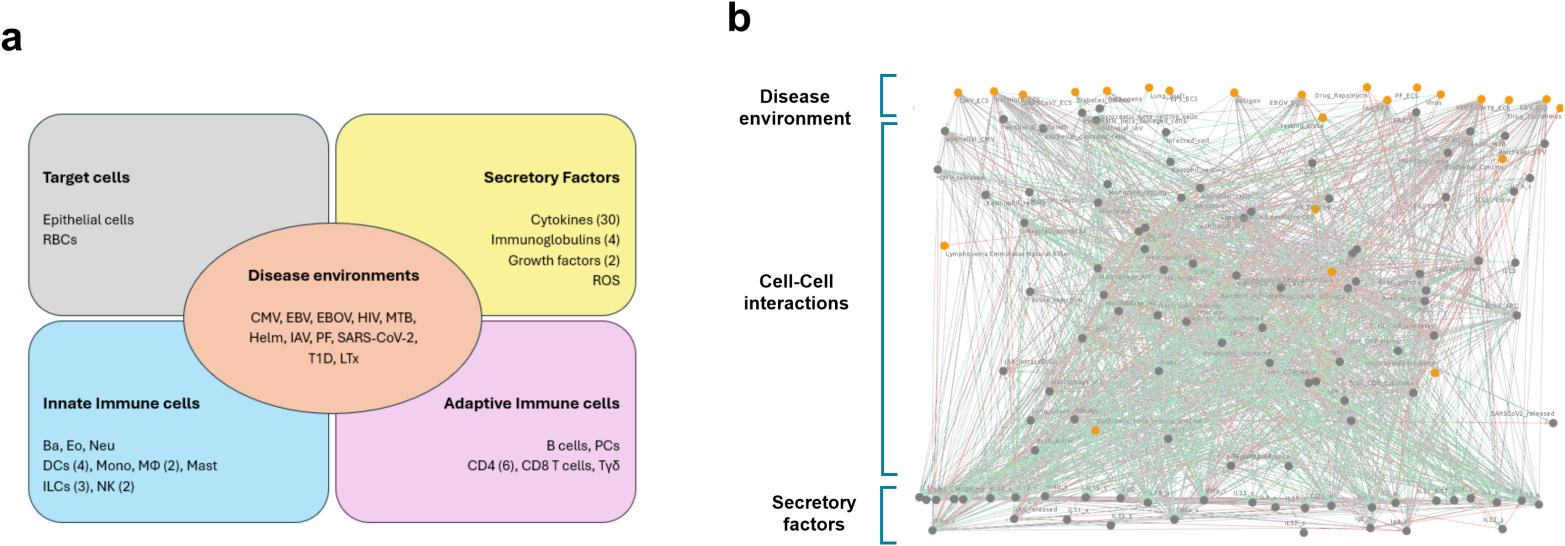
Components of the immune system model. **(a)** Overview of the cell-cell model components, with the number of subtypes per cell type mentioned in parentheses. Disease environments: Cytomegalovirus (CMV), Epstein-Barr Virus (EBV), Ebola virus (EBOV), Human Immunodeficiency Virus (HIV), Mycobacterium tuberculosis (MTB), Helminth (Hel), Influenza A virus (IAV), Plasmodium Falciparum (PF), Severe acute respiratory syndrome coronavirus 2 (SARS-CoV-2), Type 1 Diabetes (T1D), Lung transplantation (LTx). Secretory factors: Reactive oxygen species (ROS). Target cells: Red blood cells (RBCs). Innate cells: Basophils (Ba), Eosinophils (Eo), Neutrophils (Neu), Dendritic cells (DCs), Monocytes (Mono), Macrophages (MՓs), Mast cells (Mast), Innate lymphoid cells (ILCs), Natural Killer cells (NK). Adaptive cells: Plasma cells (PCs), gamma-delta T cells (Tγδ). **(b)** Network visualization of the generic template model of the human immune system in Cell Collective.

Our comprehensive model captures the complex network of signals and responses that regulate the immune response against disease conditions and comprises 126 components representing: innate and adaptive cells and their respective subtypes (e.g., T helper [Th] 1, 2, 9, 17, 22 for CD4 T cells) (**Supplementary Table 1**) and their various states (e.g., resting, naive, activated, antigen presentation); 9 pathogens; an autoimmune disease (type 1 diabetes, T1D); transplant (lung transplantation, LTx), disease/pathogen target cells (**Supplementary Table 2**); 37 secretory factors such as interleukins (ILs), immunoglobulins (Igs), growth factors, and reactive oxygen species (ROS); and 1,450 regulatory interactions among these components (**Figure 1a**). The model includes a resting state that represents immune cells in their naïve or inactivated phenotypes. For example, within the adaptive immune system, CD4⁺ T cells, CD8⁺ T cells, and B cells are initialized in their naïve states. Depending on the disease environment and immune stimuli, these cells can transition to activated phenotypes, such as helper CD4⁺ T cells, cytotoxic CD8⁺ T cells, B cells functioning as antigen-presenting cells (B-APCs), or further differentiate into memory and plasma cells. Similar methods were applied to innate cells, such as NK resting cells that can transition to NK Dim or bright in response to appropriate molecular or cellular stimuli.

In disease conditions, each disease agent is linked to target cells representing its primary site of infection. We integrate a resting state (without infection) for epithelial and red blood cells (target cells), which can transition to infected cells in response to disease environments. For example, IAV can directly infect epithelial cells, which are set up in the model as epithelial-IAV when the IAV environment is active. Therefore, to simulate the model, the resting state should always be active to simulate any disease condition (**Supplementary Figure 1E**).

To model immune cell activation, we defined regulatory rules based on experimentally validated findings. For instance, studies have demonstrated that eosinophils can be activated by either IL-5 or IL-33 (17, 18). Accordingly, in our model, the transition from resting to activated eosinophils is governed by the presence of IL-5 or IL-33 as activating signals (**Supplementary Figure 1D**). In addition, inhibitory regulatory rules were also implemented to reflect experimentally observed suppression of immune cell activation. For example, epithelial cells infected with helminths were encoded as negative inputs that prevent the activation of eosinophils, based on evidence that helminth-infected cells can inhibit eosinophil responses (19) (**Supplementary Figure 1D**).

A schematic overview of the model components, with the number of subtypes per component stated in parentheses (**Figure 1b**), is provided, along with a detailed description of each cell type and subtype included in the model, their definitions, and associated references (**Supplementary Table 1**). Our comprehensive model, built from human experimental data extracted from 449 publications, captures the intricate network of signals and responses that regulate the immune response across various disease conditions.

### Model validation: immune response to pathogens

To evaluate the accuracy and reliability of the computational model, we compared its predictions with published human *in vitro* and *ex vivo* studies and clinical observations (**Supplementary Tables 3**, **4**). We evaluated the model’s ability to reproduce innate and adaptive immune responses to individual pathogens by comparing simulated cytokine-response patterns with literature-reported biological scenarios for each pathogen represented in the model. For a validation experiment to be considered successful, the simulated cytokine behavior had to agree with the corresponding literature-derived scenario in at least 75% of cases. In total, we performed 38 validation experiments (**Supplementary Table 4**). Agreement rates were 75% for CMV, EBV, HIV, and PF; 83.3% for EBOV, IAV, MTB, and helminth infection; and 90% for SARS-CoV-2. Together, these results show that the model captures diverse features of pathogen-specific immune responses with good overall fidelity.

**Figure 2:**
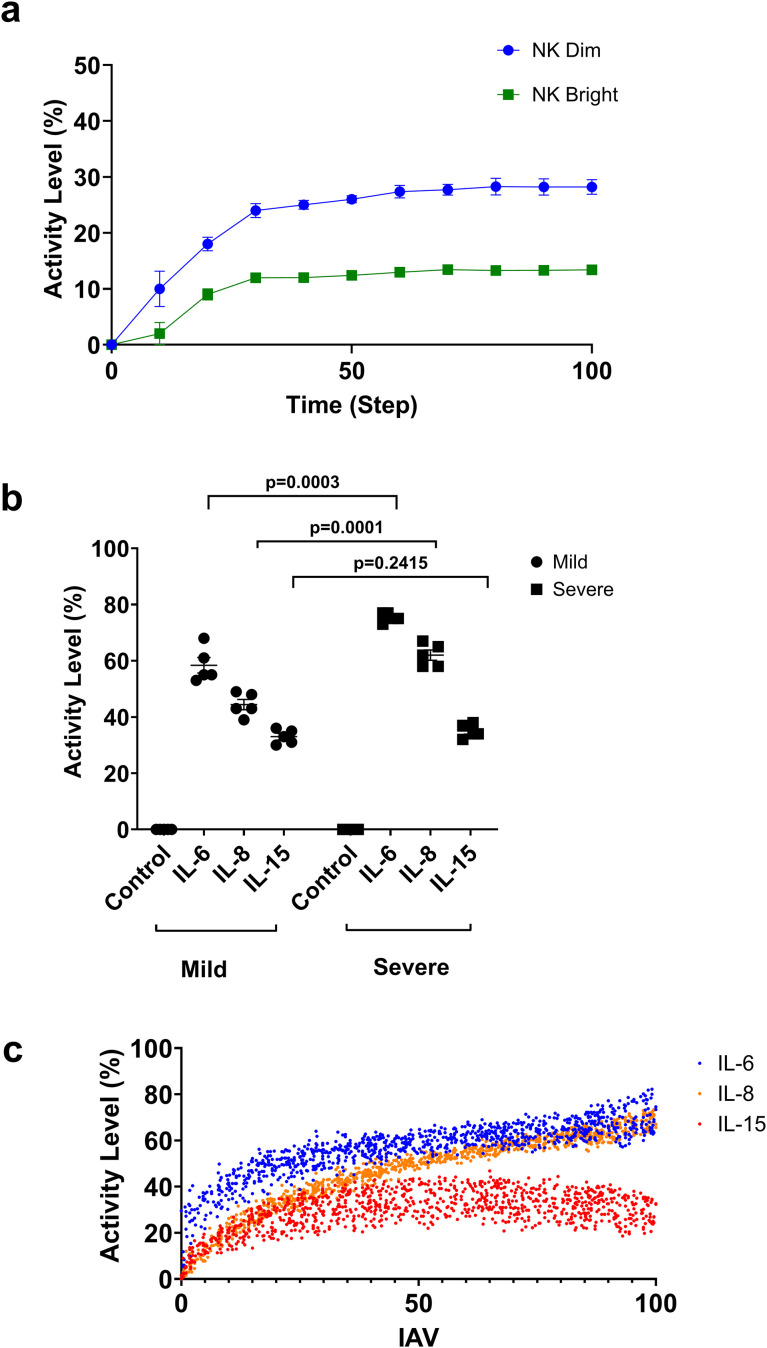
In silico validations. **(a)** Time course distribution of NK bright (green) and NK dim (blue) cells during IAV infection. **(b)** Assessment of IL-6, IL-8, and IL-15 under mild (34 activity level) and severe (95 activity level) IAV infection. (replicates n=5). Data are presented as mean ± SEM, and the *p*-value is determined by an unpaired two-tailed t-test. **(c)** Dose response curve of IL-6, IL-8, and IL-15 under varying IAV burden (replicates n= 1000).

We first evaluated the model’s ability to replicate the inherent cell responses to influenza A virus (IAV) infection. Jost et al. previously found a decline in NK bright cells and an increase in activated NK dim cells among patients infected with the H1N1 IAV strain, and our immune system model was constructed based on the experimental data from H1N1-infected patients (20). We validated the NK cell phenotype during IAV infection by running real-time simulations in our model. Results from the simulation mirror the published findings by predicting a reduction in NK bright cells during acute IAV infection (**Figure 2a**), thus demonstrating the reproducibility of the model.

We further assessed cytokine interactions during IAV infection, as cytokine activity is a key regulator of immune dynamics. Prior clinical work (Hagau et al.) reported higher levels of IL−6, IL−8, and IL−15 in patients with clinically severe versus mild influenza (21). To test whether the model reproduces these trends without conflating simulation inputs with clinical outcomes, we compared low versus high external IAV activity and measured IL−6, IL−8, and IL−15 activities. Consistent with the published findings, our model predicted that IAV severity resulted in a significant increase in IL-6 (SEM (75.4 ± .7483), p = 0.0003) and IL-8 (SEM 62 ± 1.817, p = .0001) levels during severe IAV infection, whereas IL-15 did not change significantly (SEM (35 ± 1.095). p=0.2415) (**Figure 2b**). A comparatively flat systemic IL−15 response is biologically plausible given that IL−15’s antiviral actions are often localized in the airways, and circulating IL−15 can be under−detected due to IL−15/IL−15Rα complexing; published cohorts also report non−uniform directions of association between IL−15 levels and clinical severity (22–24). **Figure 2c** adds dose-response resolution across varying IAV burden. As expected for early innate cytokines, IL−6 and IL−8 are already detectable at lower activity yet increase further (non−linearly) with higher activity, whereas IL−15 displays a shallower slope over the same range. Together, these results demonstrate the model’s ability to accurately simulate immune cell response and cytokine production following exposure to specific pathogens.

Comparing the model’s output with published human findings assesses its ability to appropriately simulate innate and adaptive immune cell dynamics during infection. As shown in **Supplementary Table 3**, our model predicted the immune responses of multiple innate and adaptive cell subtypes following exposure to select pathogens, findings supported by published literature. Specifically, we presented the reactions of several innate cells, including DCs, macrophages, monocytes, neutrophils, and NK cells, to nine pathogens, and all pathogens demonstrated activation of DCs and/or macrophages. This is attributed to their critical role as antigen-presenting cells (APCs) and as initiators of the adaptive T-cell response. In addition to DCs and macrophages, the simulation showed activation of neutrophils, NK cells, and monocytes in response to most pathogens, underscoring their critical role in pathogen clearance through phagocytosis and cytokine release (**Supplementary Tables 1**, **2**). For the adaptive response, both CD8+ T cells and the humoral response were activated under all pathogen conditions (**Supplementary Table 3**). The cytotoxic function of CD8+ T cells is essential for host defense against pathogens by eliminating infected cells, whereas the antibody response plays a crucial role in pathogen opsonization for phagocytosis and antibody-dependent cellular cytotoxicity (ADCC). Most pathogens displayed CD4+ Th1-specific immune responses, whereas helminths showed CD4+ Th2-specific immune responses. Human Immunodeficiency Virus (HIV) did not initiate CD4+ Th1- or Th2-specific immune responses, and this low activation of immune cells (e.g., CD4+ T cells) mimics the stage of disease with immunodeficiency. The model appropriately predicted that the Th1 response is primarily triggered by bacterial and viral infections, whereas Th2 is activated by parasitic infections (**Supplementary Table 3**).

**Figure 3:**
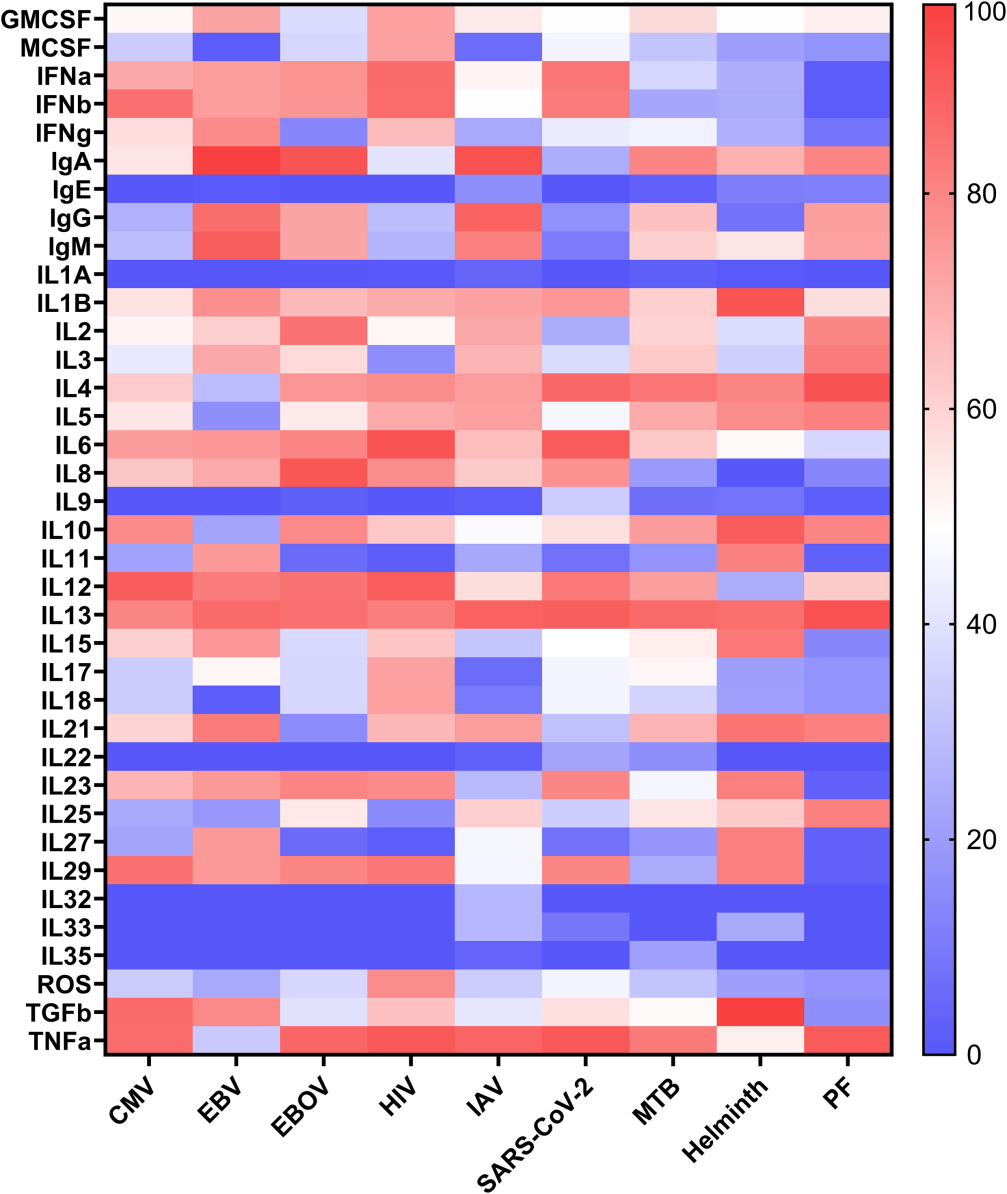
Heatmap analysis showing the secretory response to pathogen infections. The activity levels represent the average value of 100 simulation results triggered by each pathogen and were performed at 67-100% pathogen activity levels, and the resting state was performed at 67-100% activity (as a proxy to high pathogen burden).

Next, we characterized the immune response signature for each pathogen by simulating the model under single−pathogen conditions (**Figure 3**). For cytomegalovirus (CMV), the model produced a pro−inflammatory/Th1−skewed cytokine signature, with elevated IL−6, TNF−α, IL−12, IFN−γ, and IL−15, along with TGF−β engagement. These patterns are consistent with human and translational observations of CMV immunity (e.g., IL−12 -> IFN−γ and TNF−α axes; IL−6 associations; IL−15−driven NK/CD8 maintenance; CMV−induced TGF−β signaling/modulation) (25–28). For the immunoglobulin signature, IgM and IgG were detectable as expected for primary/early CMV seroconversions. IgA was present in line with mucosal humoral responses observed in milk and oral compartments, and IgE was undetectable, consistent with CMV’s typical class−switch profile where IgE is not a characteristic response and is only rarely reported (29, 30).

For Epstein-Barr (EBV), the model produced a pro−inflammatory cytokine signature with increased granulocyte-macrophage colony-stimulating factor (GM-CSF), IL-1ß, IL-6, IL-8, TNF-α, and type-I interferons (IFN-α/ß). This pattern is consistent with EBV biology: the latent membrane protein LMP1 can drive IL−6, GM−CSF, and IL−1β production in infected cells; EBV DNA/RNA engages TLR9/TLR7 in plasmacytoid dendritic cells to trigger robust type I IFN release; and EBV−encoded small RNAs (EBERs) are sensed by RIG−I and can induce type I IFN and inflammatory cytokines (31–37). Additionally, Buidiani et al. demonstrated that IFN-γ levels were elevated, while IL-4 expression was reduced in patients with EBV-associated infections. Our simulation results confirmed this trend, with an average IFN-γ activity level of 78 and an average IL-4 activity level of 29 in EBV condition (38).

For Ebola virus (EBOV), the model produced a dysregulated inflammatory immune−response signature characterized by elevated IL-1ß, IL-6, IL-12, IL-15, IFN-α/ß, TNF-α, and IL-8, accompanied by increased IL−10. This pattern aligns with human EVD cohorts, in which fatal cases show persistently high pro−inflammatory mediators together with marked IL−10, whereas survivors tend to mount more transient pro−inflammatory peaks (39–41). HIV infection in our model is characterized by pro-inflammatory (IL-1ß, IL-2, IL-6, TNF-α, IFN-α/ß/γ) and anti-inflammatory (IL-4, IL-10, IL-13) cytokines; however, some cytokines did not show any activity level, including IL-9 and IL-22, which were previously shown to be reduced during HIV progression (31). In addition, experimental and clinical data indicate IL−12/IL−18-dependent activation of NK/T−cell functions in EBOV infection, consistent with the modeled engagement of these cell types. IL−15 has also been reported in EBOV cytokine profiles, particularly around convalescence (42, 43).

For influenza A virus (IAV), which replicates in the airway and alveolar epithelium across the respiratory tract, we observed a pro−inflammatory immune−response signature in the model dominated by IL−6, TNF−α, and IL−8, with detectable IL−1β (44–46). Early type−I interferon production is present but subject to indirectly modeled viral antagonism by NS1, and a counter−regulatory IL−10 contributes to the immune response. This pattern aligns with human cohorts: IL−6 peaks early and associates with symptoms, IL−8 tracks lower−respiratory involvement, and type−I IFN is induced yet constrained; across severity strata, higher IL−2 and IFN−γ with lower IL−5/IL−17/IL−22 have been reported in severe influenza pneumonia; this is consistent with a Th1−skewed signature rather than uniform elevation of all cytokines (47–49).

Concerning SARS-CoV-2, our simulation exhibits a large panel of cytokines that overlap with those published, including IL-1ß, IL-2, IL-3, IL-4, IL-5, IL-6, IL-8, IL-10, IL-12, IL-17, IL-18, TNF-α, IFN-γ, and GM-CSF (50). This mirrors the cytokine storm and subsequent severe inflammation, immune dysfunction, and tissue damage seen in SARS-CoV-2. The model found that MTB elicits a pro-inflammatory cytokine response, increasing IL-1ß, IL-2, IL-6, TNF-α, IFN-α/ß/γ, and IL-23, and inducing some cytokines with dual functions such as IL-22, IL-27, and IL-35, which also aligns with the literature (51). Our simulations found that helminth infection prompts the production of key cytokines, such as IL-4, IL-3, IL-5, and IL-13 (**Figure 3**), that are associated with Th2 responses (**Supplementary Table 3**) (52). In contrast to other pathogens, IgG has a lower average activity level in helminth infections, consistent with prior research indicating that these antibodies are susceptible to enzymatic cleavage as a strategy to evade ADCC (53). Cytokine response to Plasmodium falciparum (PF) is mixed, pro- and anti-inflammatory, in the model, which aligns with the literature, since both pro- and anti-inflammatory responses are important in the immune response against malaria (54). Additional cytokine validations are available in **Supplementary Table 4**. Among all pro-inflammatory cytokines, IL-1ß, IL-6, and IFN-γ were identified as the most active in response to simulated infections by different pathogens. Although these cytokines play a protective role against infection, excessive secretion has been associated with increased inflammation, tissue damage, and dysfunction of immune responses in infectious diseases.

Despite extensive published knowledge of these nine pathogens, the model’s scope is limited by gaps in data on unverified interactions, particularly for specific cytokines such as IL-9, IL-32, and IL-35, which lack experimental validation, hindering a full exploration of the complex pathogen-cell-cytokine response network. In summary, the model was qualitatively consistent in cytokine and Ig production across the nine infections, effectively capturing both the shared and pathogen-specific cytokine response patterns.

### Case study 1: immune response to various coinfections

Coinfections can complicate clinical presentation, diagnosis, and treatment, often resulting in more severe symptoms and higher morbidity and/or mortality, underlining the importance of understanding the immune response during coinfection. Characterizing these responses allows identifying key differences in cytokine and immunoglobulin activity, leading to more effective targeted therapies and design strategies to prevent or manage coinfections. Furthermore, examining the immune response to coinfections provides valuable information on the complex interplay between pathogens and the host’s immune system. Understanding the immune responses to coinfections is paramount to global public health in an era of emerging and re-emerging infectious diseases.

### MTB and HIV co-infection case study

**Figure 4:**
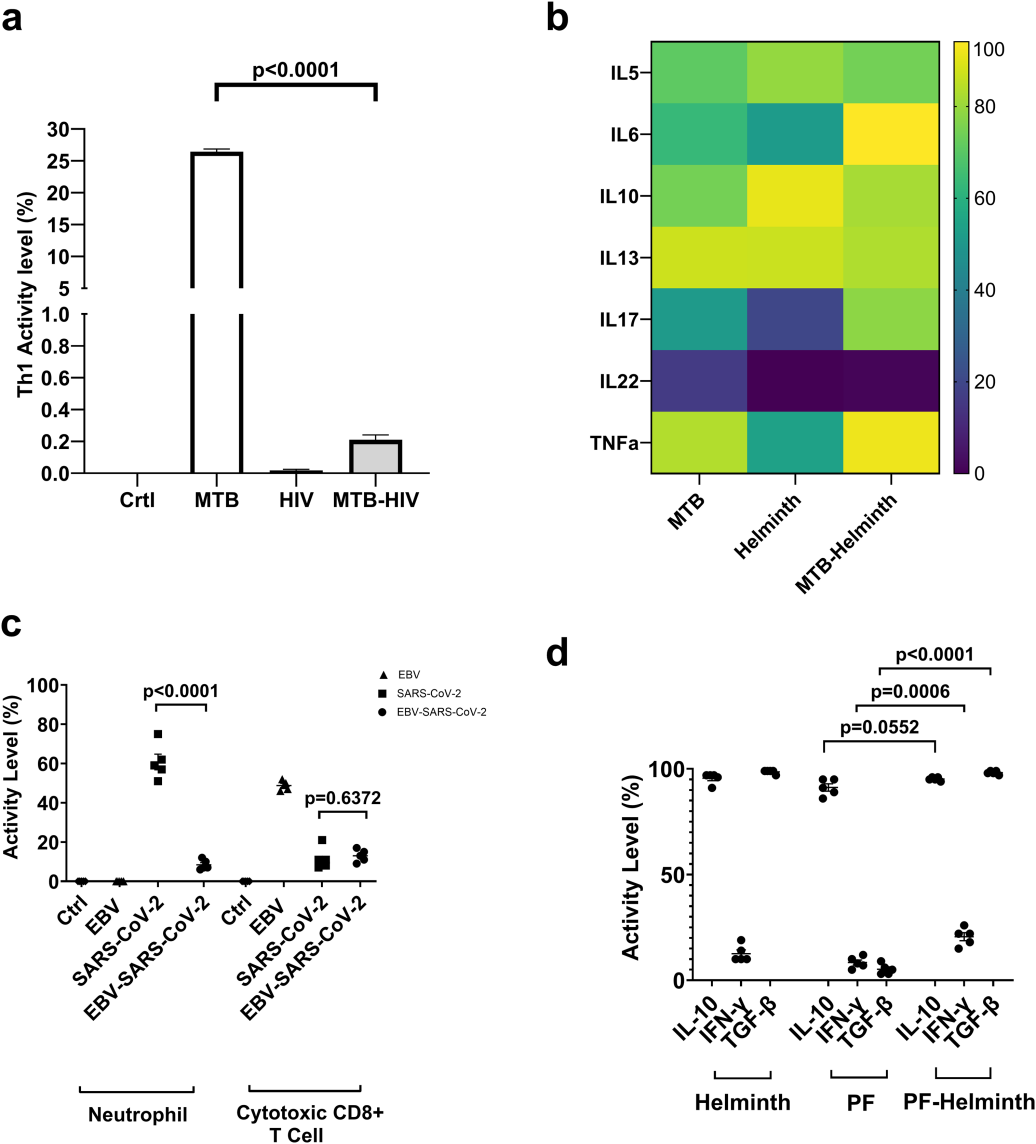
Immune response to coinfections. **(a)** Activation of CD4+ Th1 in response to MTB, HIV, and MTB-HIV coinfection (replicates n=100 simulations). **(b)** Differential cytokine response to MTB, Helminth, and MTB-Helminth coinfection. **(c)** Activity level of neutrophils and cytotoxic CD8+ T-Cells in response to SARS-CoV-2 (triangles) and coinfection of SARS-CoV-2 and EBV (Co, square) (replicates n=5). **(d)** Activity level of IL-10, IFN-γ, TGF-ß in response to PF, and PF Helminth coinfection. (replicates n=5). All data are presented as mean ± SEM, *p*-value determined by unpaired two-tailed t-test.

To address these issues, we analyzed the model’s dynamics in response to medically relevant (observed) coinfections. First, we explored the CD4+ Th1 response in MTB and HIV coinfection, using dose-response analysis, since some clinical observations showed that HIV infection induces the decline of Th1 response in coinfected patients, increasing the susceptibility to MTB infection (55). The model simulation confirmed the decrease of Th1 activity in HIV-MTB coinfection compared to MTB single infection (SEM (-26.22 ± 0.03053), p<0.0001) (**Figure 4a**).

### MTB and helminth co-infection case study

Additionally, we assessed cytokine responses in MTB-Helminth (*Strongyloides stercoralis*) coinfection. Studies showed that the synergistic effect of MTB-Helminth induces the anti-inflammatory IL-10 along with robust pro-inflammatory responses, including IL-5, IL-6, IL-13, IL-17, and IL-2 (56). Our dose-response simulation aligned well with these data, except IL-13 and IL-22, where we saw a decrease in activity level in the coinfection condition when compared to MTB alone (**Figure 4b**). In another study, Bewket et al. compared MTB single infection to MTB coinfection with helminths, both generally and with specific strains. They showed that IL-6 and TNF-α are not statistically different in MTB single infection versus coinfection with a general helminth infection (57). However, our simulation predicted an increase of IL-6 and TNF-α in MTB-Helminth coinfection (**Figure 4b**). The disagreement between simulation and literature can be explained, in part, because of our modeling of a general helminth rather than a species-specific helminth. This is supported by the findings of Bewket et al. where they showed that IL-6 and TNF-α were lower in MTB hookworm coinfection but not in any of the other measured species.

### SARS-CoV-2 and EBV co-infection case study

In the third experiment, we evaluated SARS-CoV-2 EBV coinfection using real-time simulations. Chen et al. found that coinfection of EBV and SARS-CoV-2 resulted in a non-significant decrease in neutrophils and CD8+ T-cells (58). Our simulation predicts that neutrophils significantly decrease in coinfection compared to SARS-CoV-2 alone (SEM: -52.4 ± 1.122, p <.0001) and that there is no statistical significance in cytotoxic CD8+ T-cells in coinfection when compared to SARS-CoV-2 alone (**Figure 4c**). This shows partial agreement with the literature, and the disagreement could stem from Chen et al. measuring all CD8+ T-cells while we measured only cytotoxic CD8+ T-cells.

### PF and helminth coinfection case study

Finally, we performed simulations to assess the abundance of IL-10, IFN-γ, and TGF-ß in PF and PF-Helminth coinfection. As demonstrated by Diallo et al., PF-Helminth (*Schistosoma haematobium*) coinfection was associated with higher levels of each of these cytokines in coinfected adult patients compared to those infected with PF alone (59). Consistent with these findings, our model shows IFN-γ (SEM: +12.2 ± 1.114, p = 0.0006) and TGF-ß are significantly higher in coinfection than single infection (SEM: +93 ± 0.3742, p <.0001) and higher, though not significantly (SEM: +4 ± 0.3742, p = 0.0552) levels of IL-10.

These validations demonstrate that the model replicates immune responses during coinfections in a manner consistent with published findings and can serve as a valuable tool for exploring coinfection scenarios involving immune cell-cell interactions and cytokine-mediated responses.

### Case study 2: immune response in LTx associated with CMV, EBV, and SARS-CoV-2

Lung transplant (LTx) recipients face the challenge of balancing immune suppression to prevent graft rejection while maintaining effective defenses against infections like CMV (60), EBV (61), and SARS-CoV-2 (62). The intense immunosuppressive regimens make them particularly vulnerable to CMV reactivation and severe EBV outcomes. SARS-CoV-2 poses an additional threat, with higher mortality rates and reduced lung function observed in infected transplant patients during the COVID-19 pandemic. This balance makes LTx recipients a clinically relevant context for testing how the model resolves competing signals of infection control and graft tolerance.

The LTx disease environment includes components that represent the immune response to transplanted tissue, specifically acute rejection and allograft tolerance (**Supplementary Table 5**). Activation of this environment engages a signaling network that can bias model behavior toward either graft rejection or graft acceptance. The resulting state depends on the degree of transplant-associated tissue damage sensed by the immune system and the cytokine response it elicits. In this framework, allograft tolerance, together with IL-10 activity, inhibits acute rejection and represents immune acceptance of the transplanted lung. In contrast, acute rejection suppresses allograft tolerance, indicating a shift toward immune-mediated graft injury.

**Figure 5:**
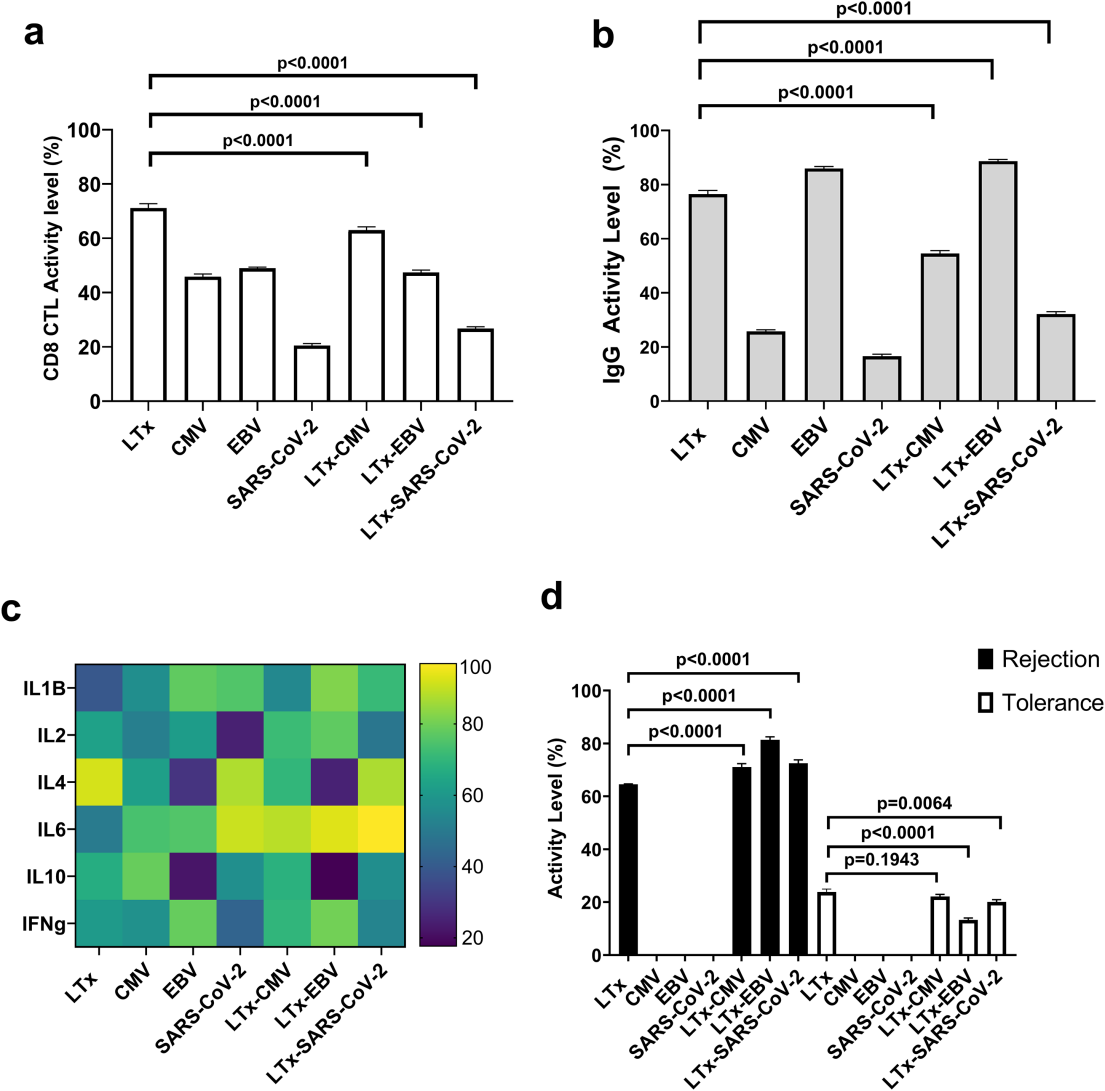
Immune response to LTx and CMV, EBV, and SARS-CoV-2 infections using dose-response analysis. **(a)** CD8+ T cell response to various single and dual infections. **(b)** Activity levels of IgG. **(c)** Cytokine profile of immune response to various pathogens and combinatorial disease conditions. **(d)** Rejection and tolerance phenotype response in single and dual conditions. The activity levels represent the average value of 100 simulation results triggered by each disease condition. Simulations were performed at 67-100% pathogen activity level. Data are presented as mean ± SEM, *p*-value determined by unpaired two-tailed t-test.

Here, we conducted a comparative *in silico* simulation of LTx conditions under three distinct viruses: CMV, EBV, and SARS-CoV-2. Previous studies have established that CD8+ T cells play dual roles, promoting rejection in LTx (63). Zaffiri et al. demonstrated that CD8+ T cell levels are higher in LTx without EBV infection compared to those with EBV (64). Others have published that the frequency of CD8+ T cells remained stable over time in LTx patients regardless of CMV infection (65). Interestingly, in SARS-CoV-2 infection, CD8+ T cell levels decrease following vaccination (66). Due to the lack of CD8+ T cell experimental validation in dual conditions of LTx and SARS-CoV-2, we validated our findings using SARS-CoV-2 vaccination data. Our simulation revealed that the CD8+ T cell response in LTx-CMV conditions decreased compared to LTx alone (SEM (-8.07 ± 1.209), p<0.0001), as well as in LTx-EBV, where CD8+ T cell activity was lower compared to LTx alone (SEM (-23.71 ± 0.8787), p<0.0001), which is consistent with previous findings. Notably, in the SARS-CoV-2 condition, the CD8+ T cell response decreased compared to LTx alone (SEM (-44.35 ± 0.6482), p<0.0001), mirroring trends observed in vaccination data (**Figure 5a**).

Next, we assessed the IgG responses under comparable environmental conditions since it is generally used as a quantitative biomarker of pathogen infection. Similar to the dual nature of CD8+ T cells in LTx, the IgG protects against infections, while also contributing to rejection by targeting donor-specific antigens. LTx patients demonstrated robust IgG responses against CMV, targeting a range of epitopes, with the response inversely correlating with viral load (67). EBV infections cause the immune system to produce IgG antibodies to multiple epitopes. Post transplant, Verschuuren et al. found that no patients had the EAD antibody but that limited levels of VCA antibody was present in the examined patient (68). Our model simulations show an increase in IgG in the LTx-EBV condition compared to LTx (SEM (12.21 ± 0.5981), p<0.0001), though how this aligns with literature findings is ambiguous since we do not have IgG epitope specificity (68). Additionally, several cohorts of lung transplant recipients receiving SARS-CoV-2 vaccines failed to mount a sufficient IgG response (69). Our findings complement the published literature showing that the IgG drop is mostly observed in SARS-CoV-2 infection (SEM (-44.28 ± 0.8504), p<0.0001) (**Figure 5b**).

We next assessed different cytokine responses for their pivotal role in promoting inflammation and tolerance. As shown in **Figure 3**, the pro-inflammatory IL-1ß and IL-6, are highly represented across most pathogens. Notably, in LTx, these cytokines have been implicated in post-transplant complications (70). Our simulation results (**Figure 5c**) aligned with clinical observations for individual conditions (LTx, CMV, EBV, and SARS-CoV-2). In dual LTx-CMV, LTx-EBV, and LTx-SARS-CoV-2 conditions, IL-6, IL-1ß and IFN-γ mirrored the patterns observed in single conditions. Additionally, a subtle increase in IL-1ß was evident in LTx-EBV compared to EBV alone.

Whitehead et al. observed elevated IL-4 levels in bronchoalveolar lavage samples from patients with acute allograft rejection compared with those without rejection (71). In our simulation, IL-4 was active in all conditions except those associated with EBV infection, where its secretion was notably reduced (29% for EBV alone and 25% for LTx-EBV), suggesting a potential dependency of IL-4 secretion on EBV infection. This observation is consistent with prior research by Buidiani et al., which demonstrated decreased IL-4 expression in EBV-associated infections (38).

Numerous studies have reported IL-10 and IL-2 in the majority of LTx patient samples, regardless of the presence or absence of complications, with occasional low expression (72). Our predictive analysis revealed a greater than 50% activity of IL-10 in all conditions except EBV and LTx-EBV and a greater than 50% activity of IL-2 in all conditions except SARS-CoV-2 and LTx-SARS-CoV-2 (**Figure 5c**). Of note, IL-10 was previously shown to increase during CMV infection (73), however, our simulation indicated that CMV infection does not significantly impact IL-10 levels in LTx coinfection.

To assess the clinical impact of the immune response in LTx, we incorporated two key phenotypes into the LTx environment: rejection and tolerance. Our simulation verified that both rejection and tolerance components are inactive in single infections (CMV, EBV, and SARS-CoV-2) (**Figure 5d**). Furthermore, our *in silico* model predicted that EBV, (SEM (16.8 ± 1.108), p<0.0001) SARS-CoV-2 (SEM (7.92 ± 1.299), p<0.0001), and CMV (SEM (6.53 ± 1.283), p<0.0001) infections elevate the likelihood of transplant rejection. Additionally, the tolerance phenotype is poor when associated with SARS-CoV-2 (SEM (-3.76 ± 0.8329), p=0.0064) and EBV (SEM (-10.55 ± 0.6807), p<0.0001). However, loss of tolerance in CMV infections is not statistically significant (SEM (-1.74 ± 0.7901), p=0.1943). Our model highlights the heterogeneous responses during viral infection and confirms the negative impact of CMV, EBV, and SARS-CoV-2 infection in LTx patients. Notably, to date, no clinical study has directly compared the outcomes of LTx patients infected with these pathogens, and our model may help to bridge these gaps.

In summary, our model demonstrated the ability to interrogate the mechanistic model in the context of lung transplantation, elucidating immune cell behavior in response to multiple infections simultaneously. Beyond the validated case studies presented here, the model generates a broader set of falsifiable predictions across monoinfection, coinfection, and transplantation contexts (**Supplementary Table 6**). These predictions include condition-specific combinations of activated immune cell states and secreted factors, as well as response patterns that emerge only in combined disease environments. Together, they define a structured hypothesis space for future experimental and clinical evaluation.

## Discussion

Digital twin (DT) technology has rapidly gained momentum across multiple sectors, including healthcare, due to its potential to simulate complex systems in a virtual setting, optimize interventions, and ultimately reduce costs and risks (74). In biology, DTs can model multifactorial processes such as tumor progression in cancer (75), optimize heart interventions in cardiology (76), and personalize glucose management in diabetes (77). These models dynamically integrate data from various sources and become increasingly accurate as real-world data updates them, enhancing their predictive potential over time.

The presented work represents a step toward developing an immune DT, focusing on the interplay between innate and adaptive immunity under multiple disease contexts. By leveraging a logical modeling framework, we built a large-scale, mechanistic model that captures direct cell-to-cell communication (e.g., antigen presentation) and secretory interactions (e.g., cytokines, immunoglobulins) across 11 disease conditions. Crucially, our validation against independent datasets - drawn from *in vitro*, *ex vivo*, and clinical observations - showed overall agreement in immune cell activation patterns, cytokine profiles, and disease-specific immune signatures. As illustrated in the multiplex cytokine analysis (**Figure 3**) and the coinfection studies (**Figure 4**), the model recapitulates known immune behaviors and identifies synergistic or antagonistic effects in complex infectious scenarios.

This comprehensive coverage highlights one of the model’s key strengths: its ability to unify multiple disease contexts and immunological processes within a single simulatable model, unlike many existing models, which are restricted to individual cell types or single diseases.

One of the major challenges in infectious diseases is the excessive secretion of pro-inflammatory cytokines, such as IL-1, IL-6, IL-18, IFN, and TNF, which can escalate into cytokine storms and cause severe tissue damage, organ failure, and high mortality rates, as observed in severe influenza and COVID-19 (78). To capture these dynamics, our multiplex cytokine analysis (**Figure 3**) examined nine distinct pathogens and revealed both unique and overlapping patterns of cytokine activity. This multi-pathogen approach highlights the model’s potential to dissect mechanisms underlying inflammatory responses and immune evasion, supporting the equilibrium between protective immunity and harmful inflammation (79, 80). Moreover, by simultaneously activating multiple disease environments, the model can investigate coinfection scenarios (81, 82), predict synergistic or antagonistic immune effects, and pinpoint strategies for reducing excessive pro-inflammatory cytokine output.

Beyond infectious settings, we used the model to simulate complex clinical contexts such as lung transplantation under various viral coinfections (**Figure 5**). Our results highlighted differential rejection and tolerance phenotypes driven by each virus, supporting the model’s ability to capture nuanced immune states. Future efforts will expand on the clinical applications by incorporating individualized biomarker and drug-regimen data and developing methods to identify infection risk profiles and optimize immunosuppressive therapies *in silico*, potentially preventing acute rejection or opportunistic infection (83, 84).

One consequential property of a large-scale mechanistic model is its capacity for systematic, biologically grounded prediction across contexts that have not yet been experimentally characterized. Unlike black-box predictive approaches, the outputs generated here are traceable to specific regulatory relationships, including cell-cell interactions, cytokine signaling logic, and pathway-level rules, that can be directly interrogated, challenged, and refined as new experimental evidence emerges. The breadth of the resulting prediction space is substantial by including eleven disease environments, more than fifty immune cell types, and thirty-seven secreted factors. The model generates structured immune-state profiles spanning single-infection responses, coinfection dynamics, and clinically complex settings such as viral challenge in the transplant context (**Supplementary Table 6**). This catalog supports experimental prioritization by identifying which cell types or cytokines are most likely to yield informative results in a given disease context, and it surfaces regulatory divergences across conditions that themselves constitute testable hypotheses, pointing to context dependencies and potential knowledge gaps that warrant targeted follow-up.

Beyond direct experimental hypothesis generation, the model’s simulatable, mechanistically labeled structure positions it as a distinctive substrate for integration with machine learning approaches. Because it can generate high-dimensional immune-state profiles across a controlled combinatorial space of disease contexts, cellular components, and regulatory perturbations, it offers a principled way to augment limited clinical datasets, support biologically informed feature engineering, and provide mechanistic grounding for approaches aimed at identifying immune biomarkers or stratifying immune phenotypes. This capacity to serve not merely as a hypothesis generator but as a structured computational prior for downstream data-driven analysis represents a particularly promising direction for future work.

Further, given the immune system’s critical role in balancing health and disease, this mechanistic model can serve as a step toward a more complex immune digital twin. Within the digital twin framework, this cellular-level model can be personalized by incorporating patient-specific data (e.g., gene expression or cytokine profiles) to constrain or initialize component activity states; however, such personalization remains limited to cellular interactions and would require integration with additional molecular and physiological layers to achieve a fully individualized immune digital twin. The presented template model can guide the expansion towards a multi-scale, multicellular framework by incorporating additional immune cell-specific sub-models of genome-scale metabolism and signal transduction. While a few such cell-type-specific molecular-level models already exist (e.g., signal transduction network models of antigen-presenting cell (85), CD4+ effector T cells (10), and macrophage (86) and constraint-based metabolic models of Th1, Th2, Th17, and regulatory T cell (87) and macrophage (88)), the majority of sub-models of other immune cells will need to be developed. Finally, we recently developed a multicellular, multi-scale computational model of CD4+ T cells that integrates physiological (ordinary differential equations), cellular (agent-based approaches), molecular (stochastic logical approach), and genome-level approaches (constraint-based approach) (12). This framework serves as a foundation for implementing additional scales atop the cellular-level generic template presented here.

### Model limitations

While the model captures a broad range of immune components and interactions, it should be viewed as a first-generation framework rather than an exhaustive representation of the human immune system. Its scope is defined by the availability of experimentally validated human data, which remains uneven across disease contexts and is particularly limited for processes such as transplantation-associated rejection and tolerance. For lung transplantation, for example, much of the existing literature relies on murine studies, patient immunoprofiling, or simplified *in vitro* systems that do not fully capture the dynamic interplay among donor-derived antigens, recipient immune responses, and the graft cytokine microenvironment. Likewise, for infectious diseases, the current model emphasizes the most well-characterized pathogens and captures generalized pathogen-specific regulatory programs rather than full strain-level diversity. As a result, the model is best interpreted as representing broad immune-response patterns rather than all possible pathogen- or tissue-specific variations.

Several additional biological dimensions also remain outside the scope of this initial version. The model does not explicitly represent immune cell homing, chemotaxis, chemokine-receptor interactions, or the structural and microenvironmental organization of infected tissues, all of which can shape local immune dynamics. To partially address this, we included pathogen-specific target cells, such as epithelial cells and red blood cells, to anchor infection at the appropriate cellular level. In addition, the present framework focuses on cellular-level immune interactions and does not yet integrate genomic, epigenetic, signaling, or metabolic layers that would further increase mechanistic resolution. These features represent important directions for future development and expansion.

Logical models have important limitations, but they remain a powerful framework for large-scale immune-system modeling. Because they are qualitative, they are not designed to estimate absolute cytokine concentrations, signaling kinetics, or other fully continuous quantitative properties. Their scope is also constrained by current biological knowledge, so unknown components or interactions may be missing. In this framework, components are represented as activity states, which simplify continuous processes, such as cytokine abundance and signaling intensity, into discrete regulatory behavior. As a result, the model does not explicitly capture fine-grained kinetic, spatial, or higher-resolution regulatory features that would require multiscale quantitative formulations. At the same time, these abstractions make logical models highly scalable, interpretable, and computationally efficient, particularly in systems where comprehensive kinetic data are unavailable. This makes them well-suited for integrating broad mechanistic knowledge, identifying key regulatory drivers and potential intervention points, and generating experimentally testable hypotheses that can guide future model refinement and multiscale expansion.

## Conclusion

In summary, this logical, mechanistic model addresses key challenges in immunological research by encompassing a broad spectrum of diseases, from single pathogens to coinfections and transplant immunology, within a single large-scale framework. The model’s availability, transparency, and accessibility in Cell Collective and SBML enable refinement and expansion to address additional scenarios, such as allergies, autoimmune conditions, and trauma. By accurately simulating pro-inflammatory dynamics, cytokine storms, and multifactorial disease interactions, it shows promise as a stepping stone toward a clinically integrated immune DT. Future work will aim to integrate multi-scale modeling approaches, encompassing genetic, molecular, cellular, tissue, organ, and physiologically based pharmacokinetics/pharmacodynamics, and organism levels. By incorporating information from these various scales, a more holistic understanding of the immune system and its role in health and disease can be developed to support novel approaches for personalized medicine.

## Methods

### Model construction and mathematical framework

We systematically searched the literature using PubMed, focusing on experiments conducted solely with human material, including PBMCs, *in vitro* cell lines, and *ex vivo* patient samples. Studies using mice and clinical trials were excluded from our manual literature mining process. We utilized multiple combinations of search terms, which included disease, secretory factor, and immune cells, as follows: “Disease X AND immune cell X”, “Disease X AND Cytokine X AND Immune cell X”, “Immune cell X AND Immune cell Y”, or “Cytokine X AND Immune cell X”.

We chose a mechanistic, logic-based model to accurately represent cellular-level interactions within the immune system in the absence of comprehensive quantitative kinetic information. Logical models use rules to describe the relationships among various components of a biological system, such as activation, inhibition, and feedback loop (89). Each component of the model can assume an active (1) or inactive (0) state at any time *t.* The activity state of the model’s internal components is determined by the regulatory mechanisms of other directly interacting components. These regulatory mechanisms are described using Boolean functions consisting of AND, OR, and NOT operator (90). We represented each immune cell type, cytokine, immunoglobulin, and disease as distinct components, with edges illustrating their interactions. To standardize component naming in the model, we used the nomenclature from the HUGO Gene Nomenclature Committee (HGNC) (91).

We built and curated the model in the web-based modeling and analysis platform, Cell Collective (15). Cell Collective is an interactive web-based platform for building, simulating, and analyzing logical and constraint-based models of biological systems (**Supplementary Figures 1A, B**). It enables researchers to construct models representing cellular networks, such as signaling pathways, gene regulatory networks, and metabolic processes, without requiring expertise in mathematical equations or coding. With its interactive interface, users can simulate dynamic behaviors, explore how various components interact, and predict system responses under different conditions. The platform is particularly useful for hypothesis testing, teaching systems biology concepts, and fostering collaboration among researchers.

All components and individual interactions used to construct the regulatory mechanisms have been annotated in Cell Collective with the exact quote from the reference literature. A total of 449 scientific publications used to build the model have been listed in the reference panel of the Cell Collective platform overview tab (**Supplementary Figure 1C**). To ensure robust evidence collection and integration, interactions were prioritized based on direct experimental evidence in human systems, with strong mechanistic validation or consistency. To address conflicting findings, we considered the strength of the experimental design and the consistency across independent studies. The model is publicly available in Cell Collective, where it can be simulated, further expanded, and downloaded in several file formats (such as SBML-qual (7), (**Supplementary Figure 1I**), text files containing the logical functions, and truth tables).

### Model simulations and analyses

We used Cell Collective for all computational simulations and analyses. Each pathogen or disease environment is represented by an external component (independent variable) in the model, whose activity level can be set by the user. Each immune cell also possesses an associated external component, allowing its initial levels to be set to represent different immune system health statuses for various conditions or subpopulations (**Supplementary Figures 1C, H**). Simulated output values range from 0 to 100% activity and represent the likelihood that a component is active under a given set of input conditions rather than an absolute molecular concentration (85) (**Supplementary Figure 1D**), and are calculated as a ratio of active and inactive states over a sliding window (89, 92). External component activity levels are unitless and provide a semi-quantitative measure of relative activity rather than a specific biological measurement (e.g., concentration). They can be specified either as fixed values or as ranges from which values are randomly sampled before each simulation, depending on the experimental design.

For coinfection simulations, multiple disease-specific external components are activated simultaneously. Interactions between pathogens are not explicitly predefined; instead, they emerge from the shared regulatory network (**Supplementary Table 7**). Immune cells and cytokines integrate concurrent signals using Boolean logic (AND, OR, NOT). This enables node-level regulatory functions to resolve potentially conflicting inputs. Consequently, context-dependent outcomes such as synergistic or antagonistic responses arise naturally depending on how signaling pathways converge on shared downstream components.

The initial condition of the model was set to resting state cellular phenotype as 1 (active), and all other components were set to zero. Simulations and analyses used probabilistic input activation states, and all components were updated synchronously as previously described (85, 93). We conducted two types of analyses: real-time and dose-response.

For real-time simulations, component activity over time was reported as the mean activity level across multiple simulations (mean ± standard error of the mean [SEM]). For real-time simulations on the Cell Collective platform (**Supplementary Figure 1E**), under the “Simulation” tab, the “External Components”, the simulations have been set to 95% to simulate the presence of specific disease external component(s) activity level, along with the resting state. For example, in **Figure 2A**, IAV activity and the resting state were each set to 95% (**Supplementary Figure 1E**), and NK dim and NK bright were initialized as active. Each in silico real-time simulation was performed in five technical replicates, which showed low variability and were sufficient to capture consistent qualitative trends in dynamic trajectories. In contrast, dose-response analyses used larger numbers of simulations to estimate averaged input-output relationships across varying levels of external component activity.

For dose-response experiments (**Supplementary Figures 1F, G**), each single-infection *in silico* experiment consisted of 100 simulations, each run for 5,000 time steps with randomly sampled external component activity levels. Output components’ activity levels were calculated as the fraction of 0’s and 1’s over the last 500 iterations (90). For dose-response analysis, the model was simulated under 67-100% pathogens’ activity levels. These simulations were designed to approximate mild (1-34%), moderate (34-67%), and severe (67-100%) infection states. The range used for the simulations is based on the highest pathogen load and the impacts on host physiology (94). For multiplex cytokine profiling, each pathogen was simulated at 67–100% activity level across 100 simulations. The resulting data were downloaded, and the average values from these simulations were calculated and visualized as a heatmap using GraphPad Prism.

As noted previously (89), and consistent with observations from the present model, the system rapidly approaches steady-state behavior, and these values are sufficient to describe its long-term (e.g., attractor-like) dynamics.

### In silico model validations

To validate the computational model, we collected independent experimental publications and reviews distinct from those employed during the model’s construction and reproduced them *via in silico* experiments (**Figure 2**; **Supplementary Table 4**). Because logical models are qualitative, model validations focus on the ability of the model to reproduce qualitative behaviors (agreement, partial agreement, or disagreement) seen in wet-lab experiments (e.g., change in the activity level of a component(s) under specific external conditions) - a standard process for logical models. Key parameters, such as the behavior of immune cell populations, pathogen activity, and secretory factor level, were cross-referenced with these independent publications to confirm consistency and reliability.

To evaluate the model’s ability to differentiate among distinct environmental conditions, we tested it by introducing one pathogen at a time. Each pathogen was activated individually in the external component of the model at 100% in real-time simulations, while the activity levels of other pathogens were monitored. A lack of any activity level in inactive pathogens was interpreted as successful environmental separation, indicating the model’s ability to correctly isolate and simulate pathogen-specific dynamics.

### Statistical analysis

Statistical analyses were performed with GraphPad Prism software using the unpaired, two-tailed Student t-test, as appropriate, to compare activity levels between groups. Results are expressed as mean ± SEM. *P* ≤ 0.05 was considered significant.

## Data Availability

The original contributions presented in the study are included in the article/**Supplementary Material**. Further inquiries can be directed to the corresponding author.
