# Supplementary material for: A comprehensive mechanistic multicellular model of the human immune system spanning 11 diseases
Source: Front Immunol. 2026 May 11;17:1732556. doi: 10.3389/fimmu.2026.1732556 (PMC13199301; doi:10.3389/fimmu.2026.1732556)
Supplement: Supplementary file 1 [file Presentation1.pptx]

## Slide 1
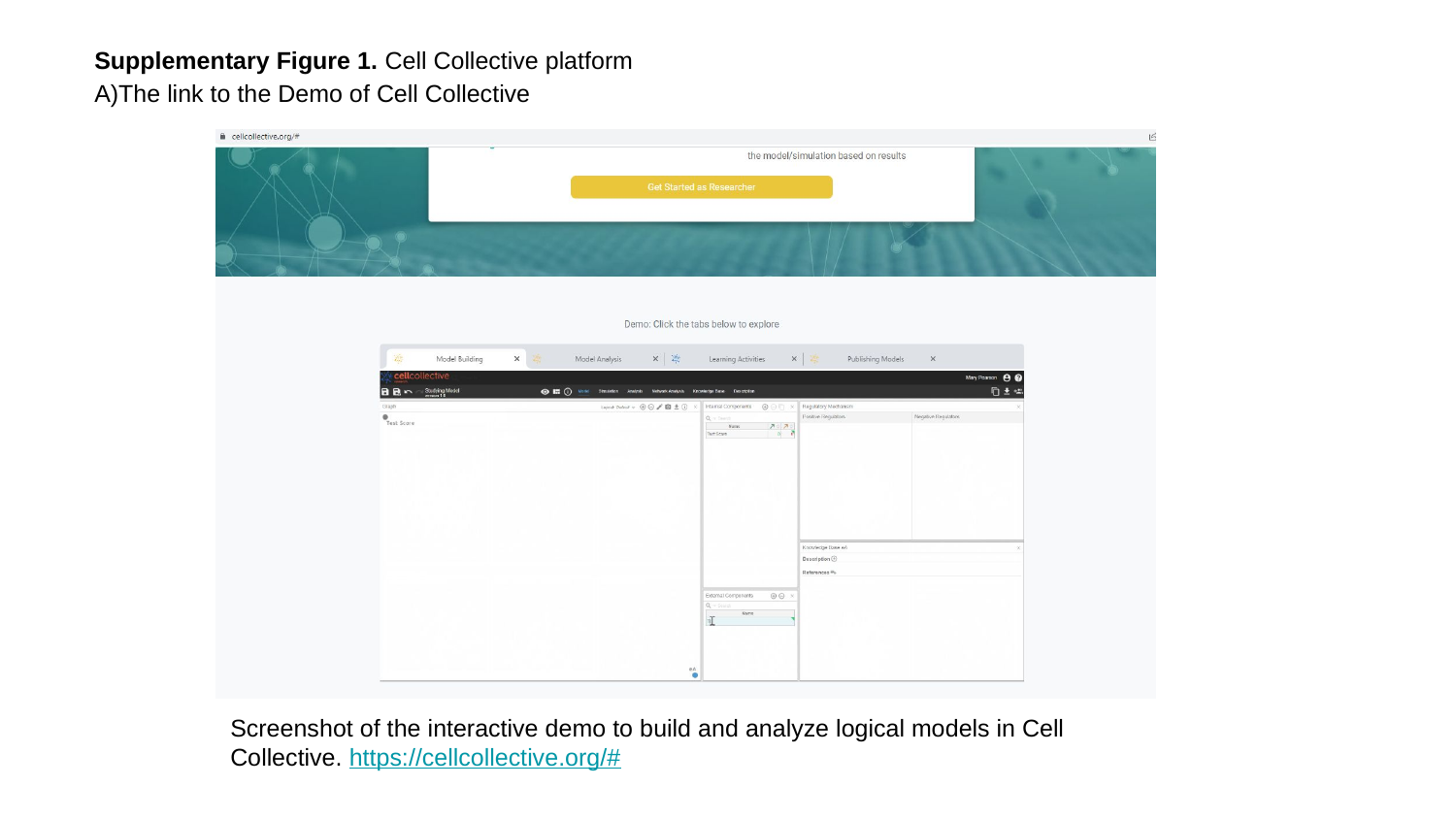

Supplementary Figure 1. Cell Collective platform
A)The link to the Demo of Cell Collective
Screenshot of the interactive demo to build and analyze logical models in Cell Collective. https://cellcollective.org/#

## Slide 2
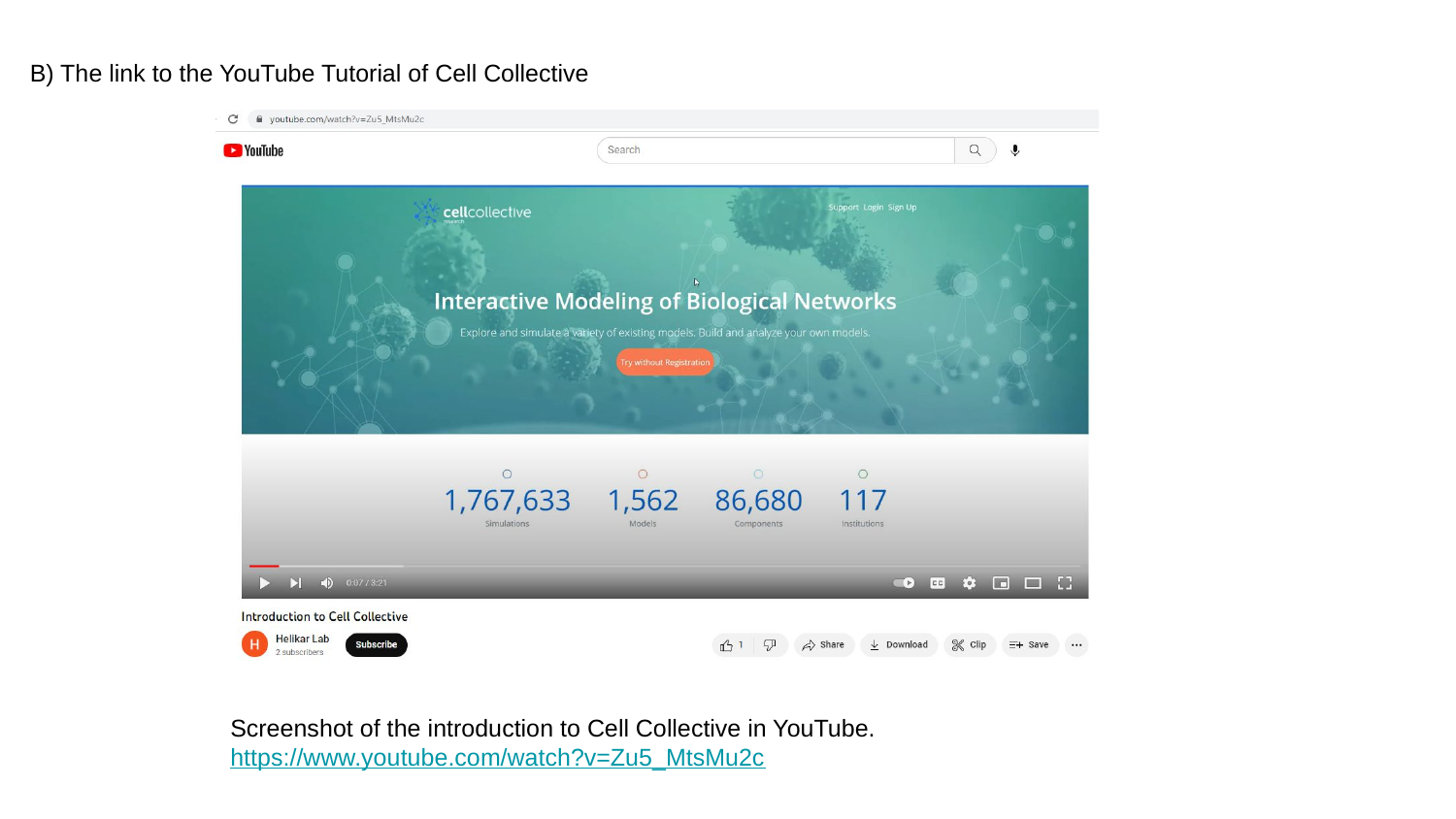

B) The link to the YouTube Tutorial of Cell Collective
Screenshot of the introduction to Cell Collective in YouTube. https://www.youtube.com/watch?v=Zu5_MtsMu2c

## Slide 3
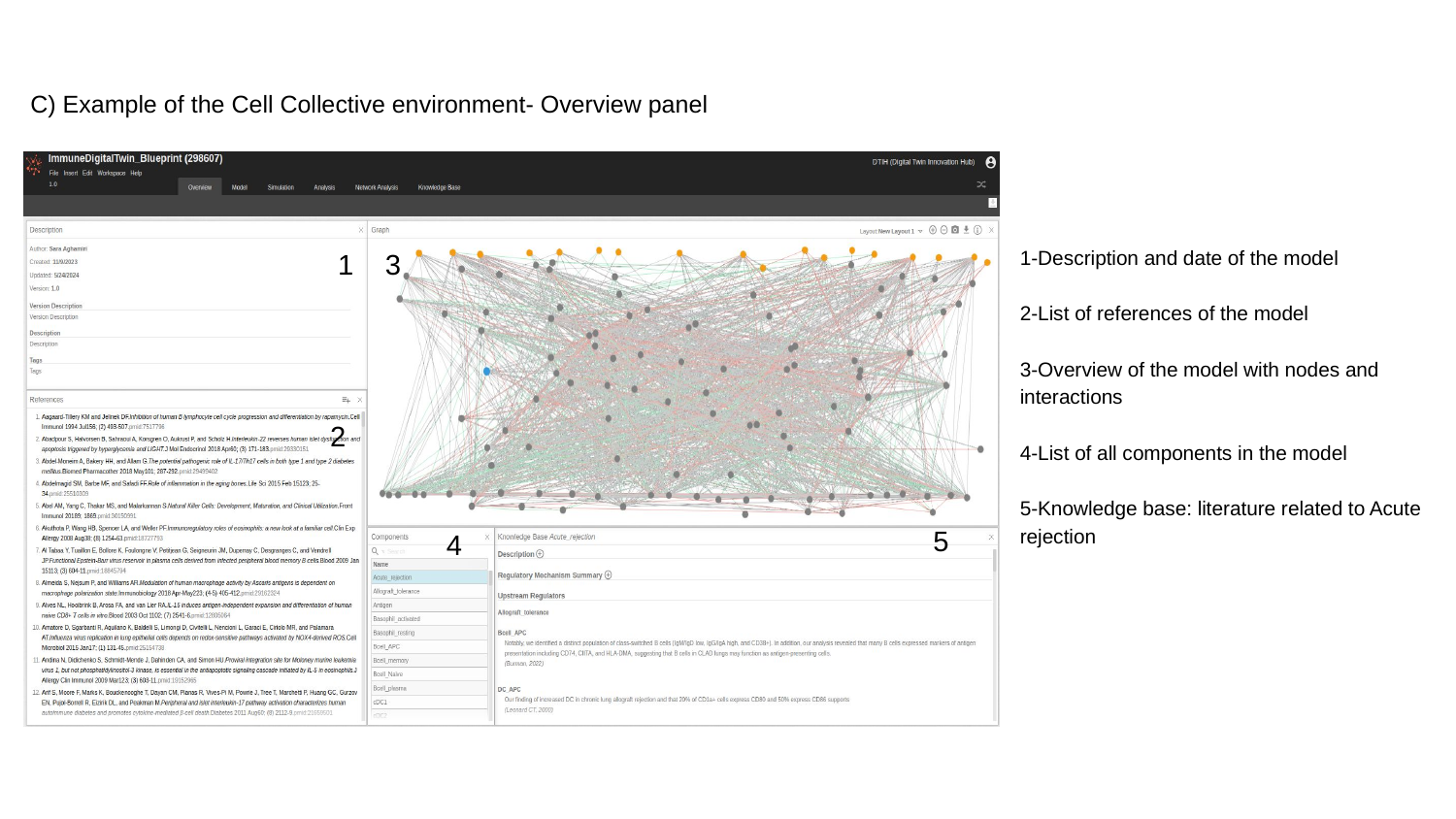

C) Example of the Cell Collective environment- Overview panel
1-Description and date of the model
2-List of references of the model
3-Overview of the model with nodes and interactions
4-List of all components in the model
5-Knowledge base: literature related to Acute rejection
1
3
2
5
4

## Slide 4
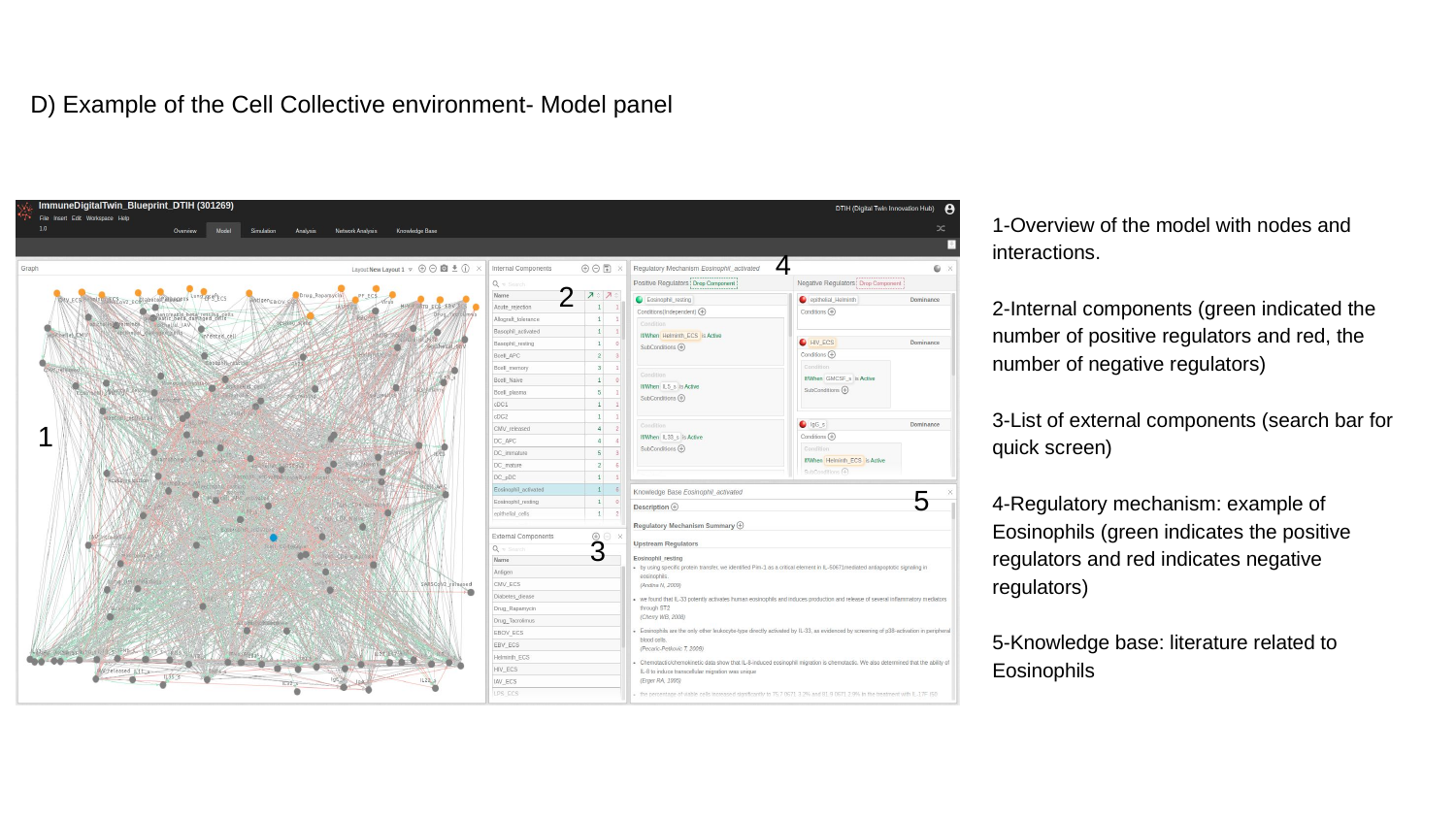

D) Example of the Cell Collective environment- Model panel
1-Overview of the model with nodes and interactions.
2-Internal components (green indicated the number of positive regulators and red, the number of negative regulators)
3-List of external components (search bar for quick screen)
4-Regulatory mechanism: example of Eosinophils (green indicates the positive regulators and red indicates negative regulators)
5-Knowledge base: literature related to Eosinophils
4
2
1
5
3

## Slide 5
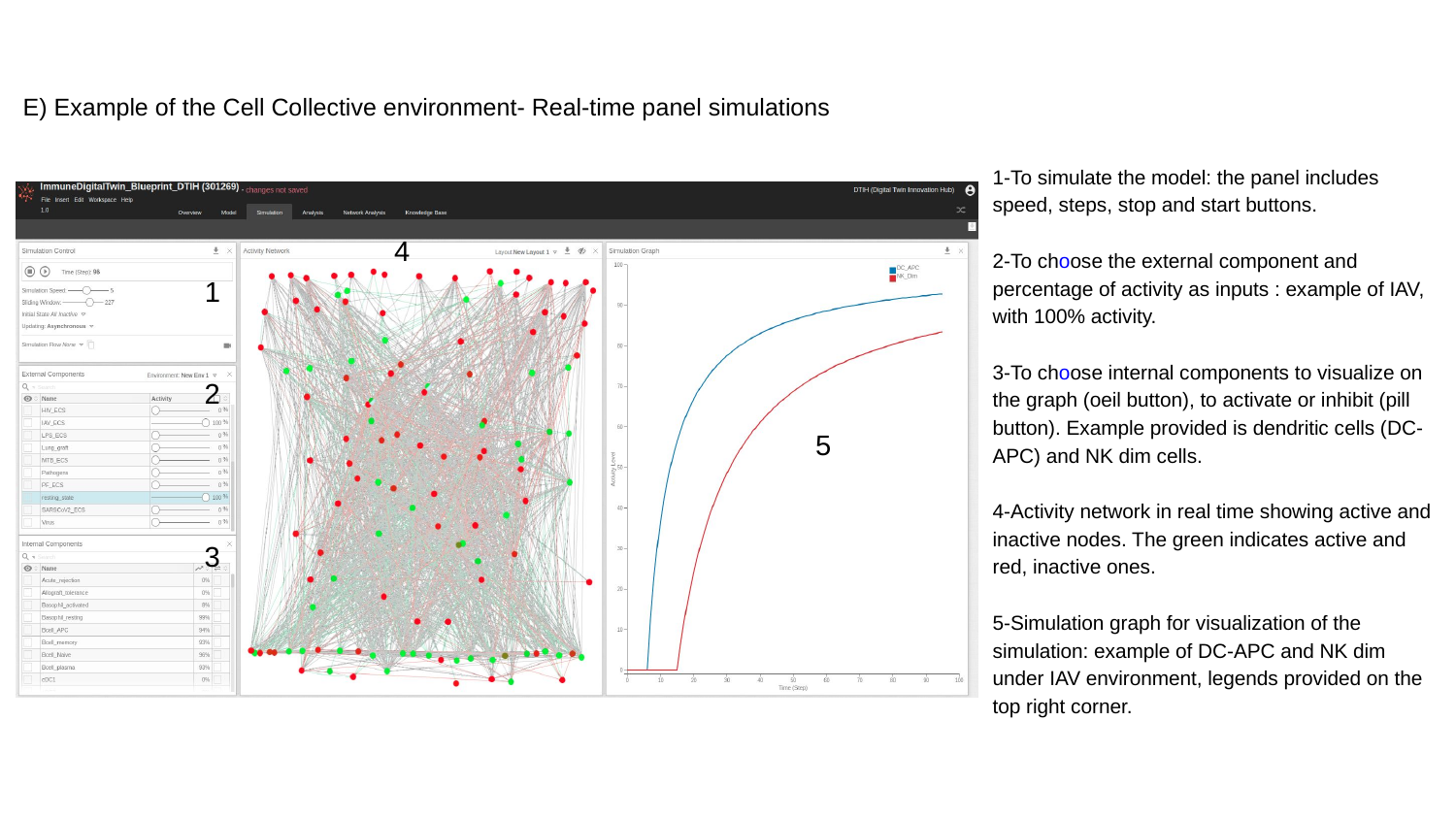

E) Example of the Cell Collective environment- Real-time panel simulations
1-To simulate the model: the panel includes speed, steps, stop and start buttons.
2-To choose the external component and percentage of activity as inputs : example of IAV, with 100% activity.
3-To choose internal components to visualize on the graph (oeil button), to activate or inhibit (pill button). Example provided is dendritic cells (DC-APC) and NK dim cells.
4-Activity network in real time showing active and inactive nodes. The green indicates active and red, inactive ones.
5-Simulation graph for visualization of the simulation: example of DC-APC and NK dim under IAV environment, legends provided on the top right corner.
4
1
2
5
3

## Slide 6
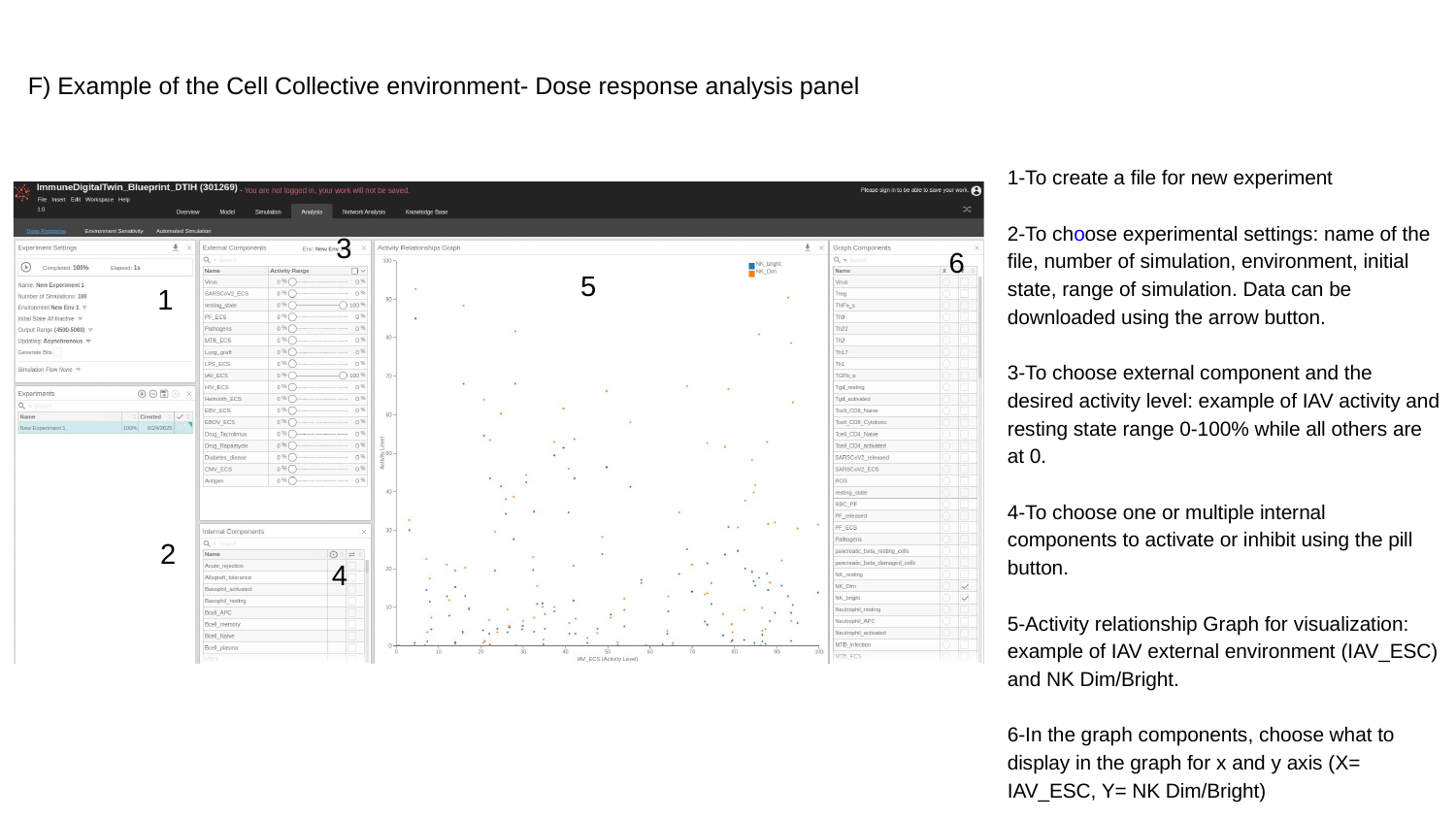

F) Example of the Cell Collective environment- Dose response analysis panel
1-To create a file for new experiment
2-To choose experimental settings: name of the file, number of simulation, environment, initial state, range of simulation. Data can be downloaded using the arrow button.
3-To choose external component and the desired activity level: example of IAV activity and resting state range 0-100% while all others are at 0.
4-To choose one or multiple internal components to activate or inhibit using the pill button.
5-Activity relationship Graph for visualization: example of IAV external environment (IAV_ESC) and NK Dim/Bright.
6-In the graph components, choose what to display in the graph for x and y axis (X= IAV_ESC, Y= NK Dim/Bright)
3
6
5
1
2
4

## Slide 7
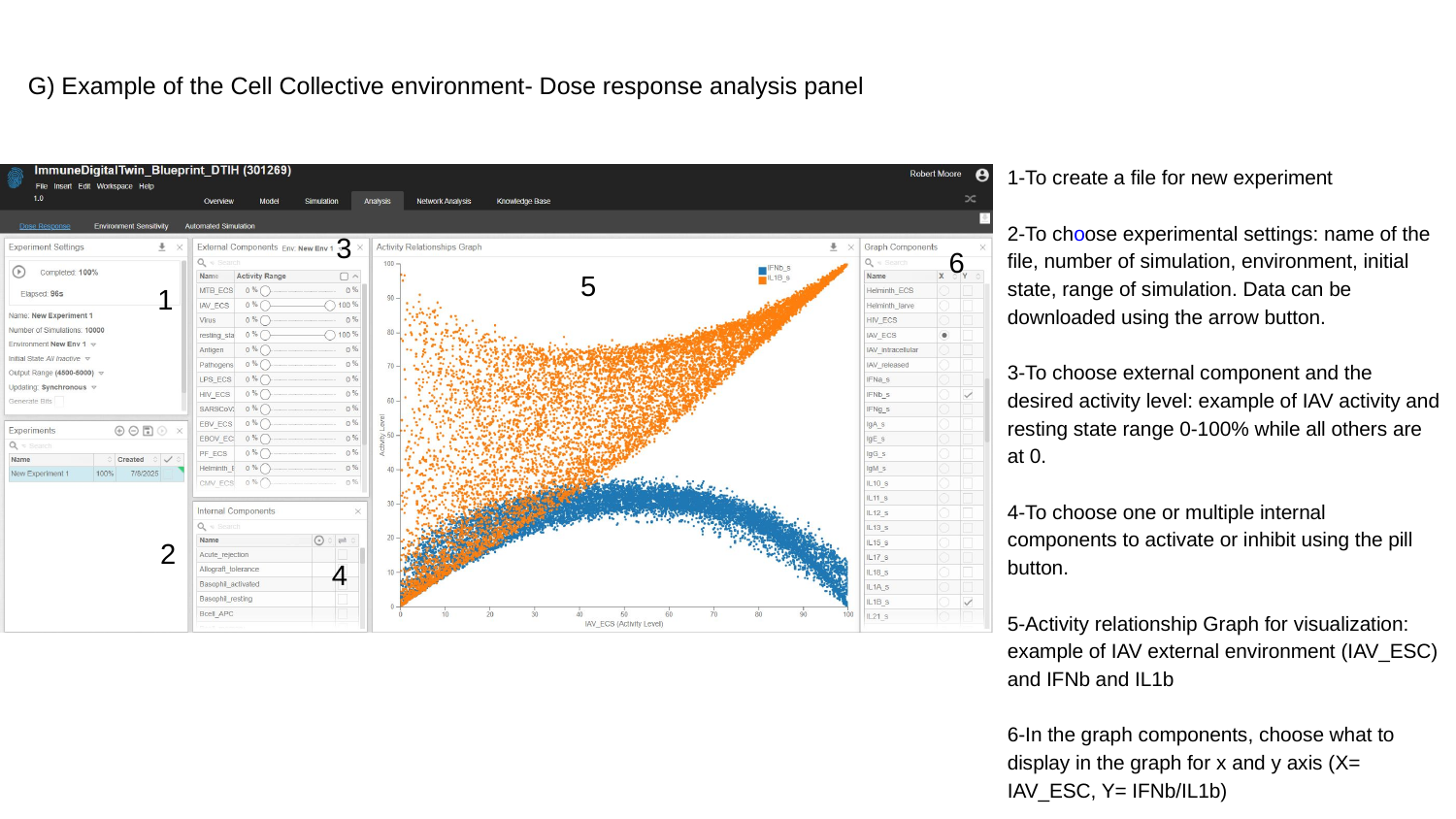

G) Example of the Cell Collective environment- Dose response analysis panel
1-To create a file for new experiment
2-To choose experimental settings: name of the file, number of simulation, environment, initial state, range of simulation. Data can be downloaded using the arrow button.
3-To choose external component and the desired activity level: example of IAV activity and resting state range 0-100% while all others are at 0.
4-To choose one or multiple internal components to activate or inhibit using the pill button.
5-Activity relationship Graph for visualization: example of IAV external environment (IAV_ESC) and IFNb and IL1b
6-In the graph components, choose what to display in the graph for x and y axis (X= IAV_ESC, Y= IFNb/IL1b)
3
6
5
1
2
4

## Slide 8
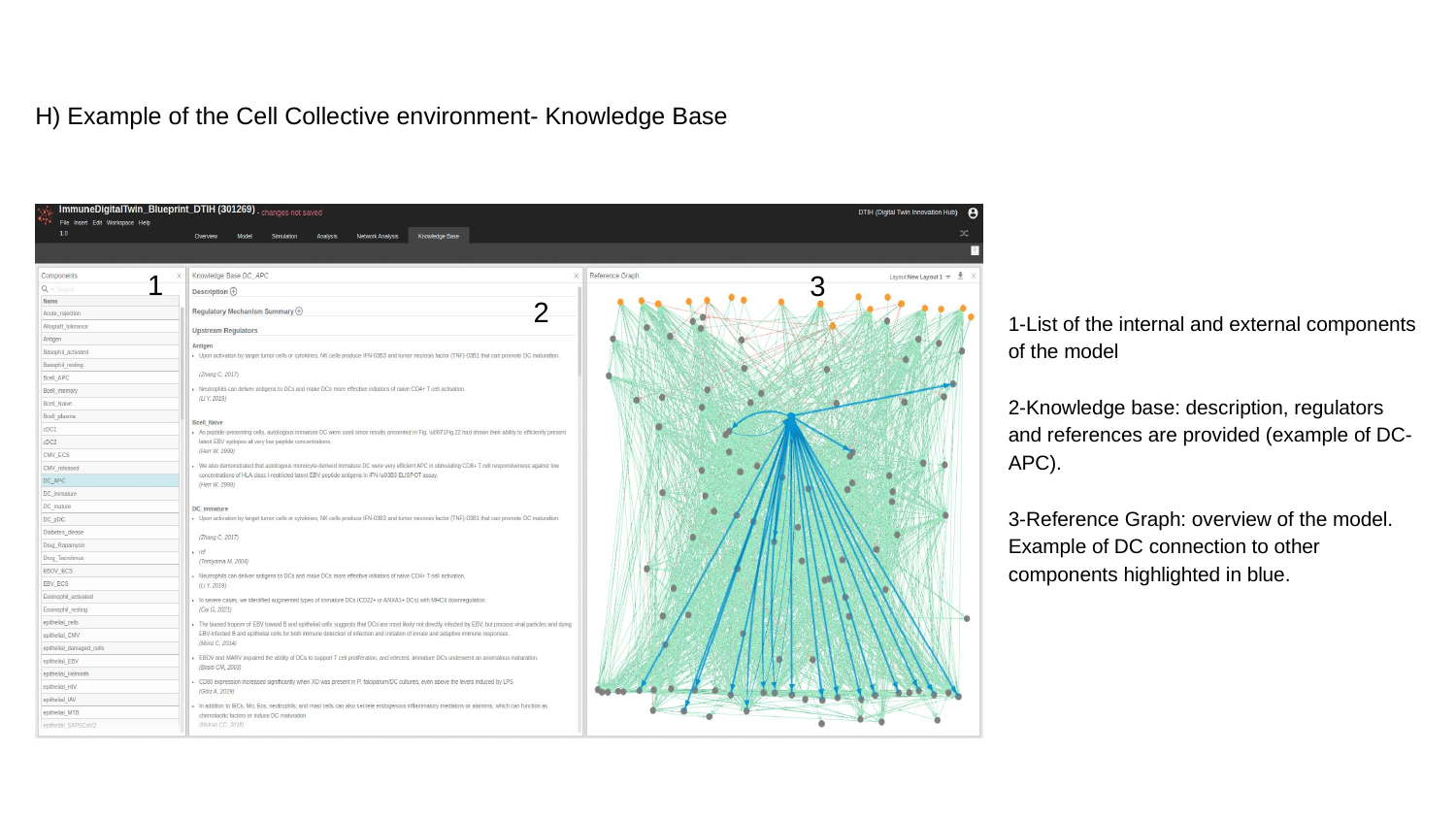

H) Example of the Cell Collective environment- Knowledge Base
1
3
2
1-List of the internal and external components of the model
2-Knowledge base: description, regulators and references are provided (example of DC-APC).
3-Reference Graph: overview of the model. Example of DC connection to other components highlighted in blue.

## Slide 9
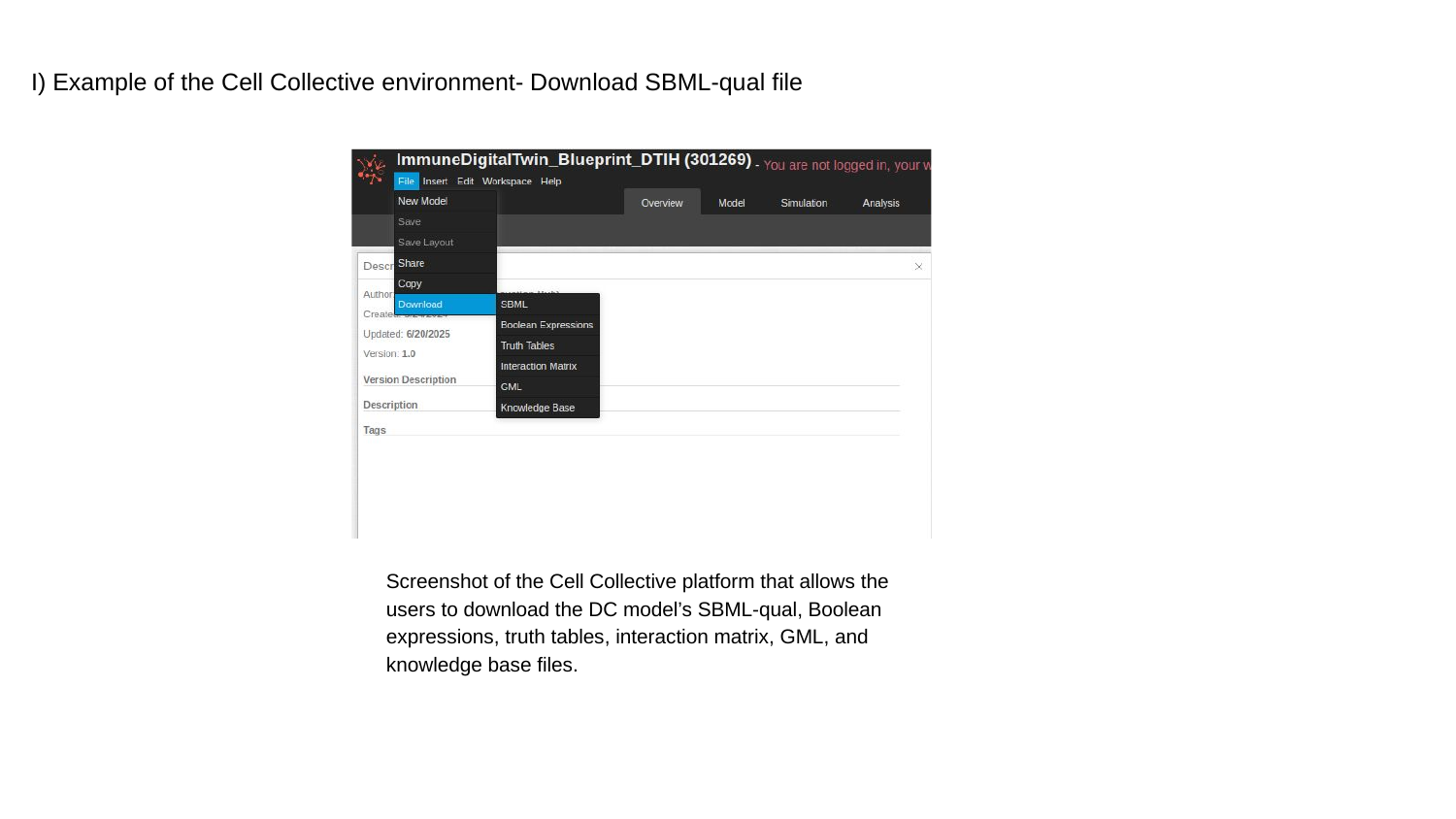

I) Example of the Cell Collective environment- Download SBML-qual file
Screenshot of the Cell Collective platform that allows the users to download the DC model’s SBML-qual, Boolean expressions, truth tables, interaction matrix, GML, and knowledge base files.
