# Supplementary material for: A comprehensive mechanistic multicellular model of the human immune system spanning 11 diseases
Source: Front Immunol. 2026 May 11;17:1732556. doi: 10.3389/fimmu.2026.1732556 (PMC13199301; doi:10.3389/fimmu.2026.1732556)
Supplement: Supplementary file 2 [file Table1.docx]

**Supplementary Information**

**Supplementary Table 1: List of cell types included in the modeled immune system.**

| **Cell Types** | **Brief Synopsis** | **Citations** |
| --- | --- | --- |
|  | **Target Cells** |  |
| Epithelial cells | These barrier-forming cells are essential for maintaining tissue integrity and preventing pathogen entry. They secrete interleukins, chemokines, and growth factors, such as IL-1, IL-6, IL-8, TGF-β (transforming growth factor-β), and GM-CSF (granulocyte-macrophage colony-stimulating factor), that recruit and activate immune cells, such as neutrophils, macrophages, and dendritic cells. | [^1^](https://www.zotero.org/google-docs/?ndR0ZJ) |
| Red blood cells (RBCs) | These cells transport oxygen, carbon dioxide, and nutrients throughout the entire body. Modifications in their structure and quantity serve as clinical indications of disease processes. | [^2^](https://www.zotero.org/google-docs/?CRQ0q9) |
| **Innate Immunity** | | |
| Basophils | These granulocytes contribute to allergic reactions and inflammation. They are activated when antigens cross-link IgE bound to their FcεRI receptors. Upon activation, basophils release histamine and other inflammatory mediators, including IL-4, IL-13, and IL-33, that increase vascular permeability and attract other immune cells, such as eosinophils, to the site of inflammation. | [^3,4^](https://www.zotero.org/google-docs/?we75Gd) |
| Dendritic cells (DCs) | These antigen-presenting cells (APCs) serve as a crucial bridge between innate and adaptive immunity. Their primary function is to capture and process antigens, which are then presented to T cells via major histocompatibility complex (MHC) molecules, initiating adaptive immune responses. Activation of DCs occurs upon recognition of pathogen-associated molecular patterns (PAMPs) or damage-associated molecular patterns (DAMPs) through pattern recognition receptors (PRRs) like toll-like receptors. Subsequently, DCs release a variety of cytokines, such as IL-12, IL-6, and IL-1, which promote T cell differentiation and activation, thus enhancing the adaptive immune response. DCs are divided into three major subtypes: monocyte-derived DCs (moDCs), conventional DCs (cDC1 and cDC2), and plasmacytoid DCs (pDCs). | [^5–7^](https://www.zotero.org/google-docs/?GDlsOz) |
| Monocyte-  derived dendritic cells (moDCs) | These DCs are derived from monocytes in response to inflammation and infection. They activate both CD8+ and CD4+ T cells through antigen presentation and the secretion of TNF-α, IL-1β, and IL-12, which are essential for driving inflammatory responses and promoting T cell polarization. | [^8^](https://www.zotero.org/google-docs/?QAN37T) |
| Conventional type 1 dendritic cells (cDC1) | cDC1 cells are crucial in initiating anti-viral immunity by efficiently cross-presenting antigens to CD4+ and CD8+ T cells. They produce high levels of IL-12, which is important for driving Th1 responses and enhancing the function of cytotoxic T cells in killing infected cells. | [^9,10^](https://www.zotero.org/google-docs/?wiX1VP) |
| Conventional type 2 dendritic cells (cDC2) | These DC subtypes initiate immune responses against extracellular pathogens, such as bacteria and fungi. They produce various cytokines, including IL-6, IL-12, IL-23, and IL-10, which help shape the immune response by promoting CD8+ T cell, Th2, Th17, and regulatory T cell responses. | [^6,9^](https://www.zotero.org/google-docs/?I9EAc2) |
| Plasmacytoid dendritic cells (pDC) | These DCs primarily sense viral infections through toll-like receptors (TLRs), particularly TLR7 and TLR9, which recognize viral RNA and DNA, respectively. Upon viral infection, pDCs rapidly produce large amounts of IFN-γ, which is responsible for the activation and proliferation of NK cells, T cells, and cDCs. | [^11,12^](https://www.zotero.org/google-docs/?kKHHRq) |
| Eosinophils | These granulocytes defend against parasitic infections and contribute to allergic inflammation. Eosinophils are activated by cytokines produced by Th2 cells, such as IL-5, and by other inflammatory mediators, such as leukotrienes. Upon activation, they release cytotoxic granules containing reactive oxygen species (ROS) and release cytokines, such as IL-4, that modulate other immune cell functions. | [^13^](https://www.zotero.org/google-docs/?G3pb1A) |
| Innate lymphoid cells (ILCs) | These cells defend against diverse infections and help initiate adaptive responses. Presented as an innate counterpart of CD4+ T helper cells, ILCs share a similar array of cytokines upon activation by epithelial- or myeloid cell-derived cytokines. They promote inflammation, mucus production, and tissue repair. ILCs comprise three subtypes: ILC1, ILC2 and ILC3. | [^14^](https://www.zotero.org/google-docs/?YsUUDq) |
| Type 1 innate lymphoid cells (ILC1s) | These cells are crucial in early defense against viral infection, as they can directly kill infected cells by producing cytotoxic mediators, including granzymes and perforins. ILC1s secrete Th1 cytokines that mirror those produced by CD4+ T cells, including the production of IFN-γ and TNF-α. | [^15^](https://www.zotero.org/google-docs/?QIOqt6) |
| Type 2 innate lymphoid cells (ILC2s) | ILC2s are the innate counterparts of Th2 cells and protect against parasite infections. They secrete cytokines similar to Th2 cells, including IL-4, IL-13, and IL-5, that promote the recruitment of eosinophils and mast cells and facilitate the differentiation of M2 macrophages and plasma cells. | [^16^](https://www.zotero.org/google-docs/?dThC6j) |
| Type 3 innate lymphoid cells (ILC3s) | Presented as sentinel cells in the mucosa, these cells mainly defend against bacteria and fungi. By secreting similar cytokines to Th17 cells, including IL-17, IL-22, and GM-CSF, ILC3s promote neutrophil recruitment, plasma cell differentiation, and myeloid activation. | [^17^](https://www.zotero.org/google-docs/?zgQeyK) |
| Macrophages | These phagocytic cells engulf and clear pathogens, remove cellular debris, and produce cytokines that regulate inflammation and immune responses. Macrophages can be activated by PAMPs, DAMPs, or cytokines, such as IFN-γ (interferon-γ) and GM-CSF. Upon activation, macrophages release a diverse array of cytokines, including IL-1, IL-6, TNF-α (tumor necrosis factor-α), IL-10, and IL-12, as well as other factors like nitric oxide, ROS, and inducible nitric oxide synthase (iNOS). These molecules collectively modulate immune cell activation and differentiation, thereby orchestrating immune responses. Macrophages comprise two main subtypes: M1 and M2 macrophages. | [^18^](https://www.zotero.org/google-docs/?x5pkjy) |
| M1 macrophages | M1 macrophages are classified as pro-inflammatory cells and produce a variety of pro-inflammatory cytokines, such as IL-1, IL-6, IL-12, and TNF-α, upon microbial infection or in the presence of IFN-γ in the milieu. These cytokines create an inflammatory milieu that enhances the recruitment and activation of other immune cells, such as T cells and NK cells. Additionally, the high levels of reactive oxygen species (ROS) and nitric oxide (NO) produced by M1 macrophages contribute to pathogen clearance and the immune response. | [^19^](https://www.zotero.org/google-docs/?R49aX1) |
| M2 macrophages | In contrast to M1 macrophages, M2 macrophages are anti-inflammatory cells that resolve inflammation through the production of anti-inflammatory cytokines. The cytokines produced by M2 macrophages, including IL-4, IL-13, IL-10, and TGF-β, foster an anti-inflammatory environment that inhibits the activity of pro-inflammatory cells and promotes the resolution of inflammation. M2 macrophages are essential in tissue repair, homeostasis, and wound healing after an inflammatory response. | [^19^](https://www.zotero.org/google-docs/?slldcZ) |
| Mast cells | These cells play a role in allergic reactions and inflammation. Upon crosslinking of the IgE-antigen complex to the surface receptor FcεRI, mast cells release histamine, tryptase, and other mediators. Mast cells also produce cytokines, such as TNF-α, IL-4, IL-5, IL-6, and IL-13, which modulate the function of other immune cells, including T cells and DCs. | [^20^](https://www.zotero.org/google-docs/?x4yefg) |
| Monocytes | These circulating cells differentiate into macrophages or DCs upon entering tissues. They participate in phagocytosis and release cytokines, such as IL-1, IL-6, TNF-α, and IL-10, which regulate inflammation and immune responses. Monocytes can be activated by PAMPs, DAMPs, or cytokines, such as GM-CSF and M-CSF (macrophage colony-stimulating factor). | [^21^](https://www.zotero.org/google-docs/?arWxEj) |
| Neutrophils | These phagocytic cells are the first responders to infection and tissue damage. They release antimicrobial peptides, proteases, and ROS to destroy pathogens. Neutrophils are rapidly recruited to sites of infection or inflammation by chemokines, such as IL-8 and MIP-1ɑ, and other inflammatory mediators produced by epithelial cells, endothelial cells, and other immune cells. | [^22^](https://www.zotero.org/google-docs/?Lzn8Ar) |
| Natural Killer (NK) cells | These cytotoxic cells recognize and directly kill virus-infected and cancerous cells without prior sensitization. Activation of NK cells occurs through various mechanisms, including cytokine stimulation (e.g., IL-12, IL-15, and IL-18) and recognition of stress-induced ligands on target cells. Upon activation, NK cells not only perform cytotoxic functions but also secrete immunomodulatory cytokines, such as IFN-γ. The cytokine production by NK cells promotes Th1 responses and augments the cytotoxic activity of CD8+ T cells. | [^23,24^](https://www.zotero.org/google-docs/?EFedDu) |
| NK bright cells | These cells demonstrate an immunomodulatory role by secreting both pro- and anti-inflammatory cytokines, including IFN-γ, TNF-α, IL-10, and GM-CSF. NK bright cells are described as immature and with low cytotoxic ability, but upon infection, they can differentiate into a mature NK dim cell that displays higher cytotoxic potential. | [^23,24^](https://www.zotero.org/google-docs/?tPKYTT) |
| NK dim cells | These cells can directly induce apoptosis of infected cells by releasing cytotoxic granules and initiating antibody-dependent cellular cytotoxicity (ADCC). NK dim cells can also secrete IFN-γ and TNF-α, but to a lesser extent than NK bright cells. These cytokines play significant roles in promoting immune cell recruitment and enhancing antigen presentation capabilities of DCs and macrophages. | [^23,24^](https://www.zotero.org/google-docs/?gwnf0N) |
| **Adaptive Immunity** | | |
| B cells | B cells are integral to the adaptive immune system, expressing membrane-bound antibodies called immunoglobulins (Ig) (such as IgM, IgA, IgE, and IgG classes) that target and neutralize specific pathogens. Additionally, activated B cells can serve as antigen-presenting cells for T cells, further enhancing the coordination of immune responses. | [^25^](https://www.zotero.org/google-docs/?AXtP08) |
| Plasma cells  (PCs) | Upon encountering their specific antigen, B cells differentiate into antibody-secreting PCs, a process mediated by CD4+ T cells. This immunological synapse relies on the interaction between CD40 ligand (CD40L) on T cells and CD40 on B cells, complemented by the release of cytokines, such as IL-4, IL-6, and IL-21. | [^25^](https://www.zotero.org/google-docs/?v1XoWI) |
| CD4+ T cells | Upon activation by DCs presenting antigens via MHC II, naive CD4+ T cells can differentiate into various Th cell subsets, such as Th1, Th2, Th9, Th17, Th22, and regulatory T cells (Tregs). This differentiation is influenced by the cytokine milieu produced by other immune cells, such as DCs, macrophages, and NK cells. | [^26,27^](https://www.zotero.org/google-docs/?ExchsZ) |
| T helper 1 (Th1) cells | These CD4+ T cells are involved in cell-mediated immunity against intracellular pathogens, such as viruses and some bacteria. They produce IFN-γ and TNF-α, which promote macrophage activation and enhance the cytotoxic activity of CD8+ T cells and NK cells. Th1 cell differentiation is induced by IL-12 and IFN-γ, which are released by antigen-presenting cells, such as DCs and macrophages, in response to intracellular pathogens. | [^26,28^](https://www.zotero.org/google-docs/?NTuUhS) |
| T helper 2 (Th2) cells | These CD4+ T cells are involved in humoral immunity against extracellular pathogens, such as parasites. They produce IL-4, IL-5, and IL-13, which promote B cell differentiation into plasma cells, eosinophil activation, and mast cell activation. Th2 cell differentiation is induced by IL-4, which DCs, basophils, or mast cells can release. | [^28,29^](https://www.zotero.org/google-docs/?gKFsfs) |
| T helper 9 (Th9) cells | These CD4 + T cells are characterized by their ability to produce IL-9, a cytokine involved in various immune responses. Th9 cells play a role in immune-related diseases such as inflammation, allergy, and parasite infection. | [^30^](https://www.zotero.org/google-docs/?UyqoW1) |
| T helper 17 (Th17) cells | These CD4+ T cells play a role in defending against extracellular bacterial and fungal infections. They produce cytokines, such as IL-17A, IL-17F, IL-21, and IL-22, which stimulate the production of antimicrobial peptides and the recruitment of neutrophils. Th17 cell differentiation is induced by the cytokines IL-6, TGF-β, IL-1, and IL-23. | [^28,31^](https://www.zotero.org/google-docs/?V0FU0o) |
| T helper 22 (Th22) cells | These cells are named after the main cytokine that they produce, IL-22. These CD4+ T cells are involved in immune responses at barrier surfaces, such as the skin and mucosal membranes, and contribute to defense against certain infections, including HIV and influenza. Additionally, Th22 cells have been implicated in various inflammatory and autoimmune conditions. | [^32^](https://www.zotero.org/google-docs/?KzXOE0) |
| Regulatory T cells (Treg) | These CD4+ T cells play a critical role in maintaining immune tolerance and preventing autoimmunity. They suppress the activation and function of other immune cells, including B cells, CD4+ T cells, CD8+ T cells, and DCs, through various mechanisms, such as the secretion of immunosuppressive cytokines (e.g., TGF-β and IL-10) and direct cell-cell interactions. Treg differentiation is induced by TGF-β and IL-2. | [^26,28^](https://www.zotero.org/google-docs/?W3GMuI) |
| CD8+ cytotoxic T cells | These cytotoxic T cells recognize and kill virus-infected and cancerous cells by releasing cytotoxic granules containing perforin and granzymes. CD8+ cytotoxic T cell activation requires antigen presentation by MHC I molecules on infected or abnormal cells, as well as help from CD4+ T cells via cytokines such as IL-2 and IFN-γ. | [^33,34^](https://www.zotero.org/google-docs/?iOIZBR) |
| Gamma-  delta (γδ) T cells | These cells represented a minor subset of T cells, capable of presenting antigens like innate cells. These cells have dual functions: either maintaining immunosuppression or activating inflammation, depending on the cytokines they produce, including IL-4, IL-17, IL-21, IL-22, GM-CSF, and IFN-γ. | [^35^](https://www.zotero.org/google-docs/?XgbIln) |

**Supplementary Table 2: List of disease conditions of the modeled immune system.**

| **Disease Environments** | **Brief Synopsis** | **Citations** |
| --- | --- | --- |
| Cytomegalovirus (CMV) | Cytomegalovirus (CMV) triggers an innate response involving DCs and NK cells, leading to the release of type I interferons to regulate the early stages of infection. During the persistence phase, CD4+ T cells, CD8+ T cells, and B cells, primed by NK cells and DCs, orchestrate a vigorous and enduring immune response to restrain high viral loads. Despite this response, CMV can establish a latent state within the host, posing a risk of reactivation and reinfection, particularly when the immune system is compromised. | [^36^](https://www.zotero.org/google-docs/?EWxUoz) |
| Epstein-Barr virus (EBV) | Epstein-Barr virus (EBV) primarily infects B cells, which can be eliminated by NK cells, effector CD4+ T cells, and CD8+ T cells, thereby controlling the initial phase of infection and maintaining viral latency. However, sporadic reactivation may occur, evading immune surveillance despite the presence of long-lasting CD8+ and CD4+ T cell responses. | [^37,38^](https://www.zotero.org/google-docs/?eiO1NV) |
| Ebola virus (EBOV) | Ebola virus (EBOV) triggers a multifaceted immune response, where innate defenses mobilize macrophages, DCs, and NK cells to release proinflammatory cytokines such as TNF-α, IL-6, and IFN-α/β. In the adaptive arm, CD8+ T cells directly target infected cells, while CD4+ T cells secrete key cytokines like IFN-γ and IL-2. Additionally, plasma cells produce neutralizing antibodies to counteract the virus. | [^39,40^](https://www.zotero.org/google-docs/?D4cu2l) |
| Human immunodeficiency virus (HIV) | The immune response against Human Immunodeficiency Virus (HIV) comprises both innate and adaptive components. In the innate immune response, DCs, NK cells, and macrophages produce IFN-α/β and other cytokines. Adaptive immunity involves CD8+ T cells, which directly target infected cells, and CD4+ T cells, which secrete cytokines such as IFN-γ and IL-2. Plasma cells contribute by producing neutralizing antibodies. However, HIV's high mutation rate enables it to evade the immune system, posing a significant challenge for effective immune responses. | [^41^](https://www.zotero.org/google-docs/?UXBxHm) |
| Influenza A virus (IAV) | The immune response against influenza A virus (IAV) commences with the activation of the innate immune system, comprising macrophages, DCs, and NK cells, which release cytokines such as IFN-α/β, TNF-α, and IL-6. Adaptive immunity then follows, with CD4+ T cells producing cytokines like IFN-γ and IL-2 to orchestrate the immune response, while CD8+ T cells target and eliminate infected cells. Additionally, plasma cells produce neutralizing antibodies that specifically recognize and bind to viral surface proteins, such as hemagglutinin and neuraminidase, aiding in viral clearance. | [^42^](https://www.zotero.org/google-docs/?aT4G9e) |
| Mycobacterium tuberculosis (MTB) | The immune response to Mycobacterium tuberculosis (MTB) involves macrophages engulfing the bacteria and producing cytokines such as TNF-α, IL-1, and IL-6. CD4+ T cells produce IFN-γ and IL-2, activating macrophages to eliminate intracellular bacteria. Simultaneously, CD8+ T cells target and eradicate infected cells. Although plasma cells produce antibodies, the precise role of plasma cells in MTB immunity remains incompletely understood. | [^43,44^](https://www.zotero.org/google-docs/?GF2pwu) |
| Severe acute respiratory syndrome coronavirus 2 (SARS-CoV-2) | COVID-19, caused by the SARS-CoV-2 virus, induces the activation of neutrophils, monocytes, and macrophages. In severe cases of COVID-19, inflammation disrupts both innate and adaptive immune responses, leading to excessive cytokine release, including IL-6, IL-1β, and TNF-α. These cytokines are implicated in the development of clinical complications associated with the disease. | [^45,46^](https://www.zotero.org/google-docs/?2xpTiy) |
| Helminth | These parasitic worms trigger a multifaceted immune response. Initially, the innate immune system responds by activating group 2 innate lymphoid cells (ILC2) and eosinophils, which release a cascade of cytokines including IL-4, IL-5, IL-9, and IL-13. These cytokines play crucial roles in orchestrating defense against helminth infections by promoting mucus production, smooth muscle contraction, and recruitment of additional immune cells to the site of infection. Subsequently, the adaptive immune system mounts a Th2 response, characterized by CD4+ T cells secreting cytokines similar to those produced by innate immune cells. | [^47,48^](https://www.zotero.org/google-docs/?A9q4Sm) |
| Plasmodium falciparum (PF) | The pathogen Plasmodium falciparum (PF), the causative agent of severe malaria, elicits a complex immune response. In the innate arm, macrophages and neutrophils phagocytose infected RBCs to curb parasite proliferation. DCs and NK cells contribute by releasing key cytokines such as IFN-γ and TNF-α, which aid in parasite clearance. The adaptive immune response is orchestrated by CD4+ T cells, which produce a spectrum of cytokines, including IFN-γ, IL-2, and IL-10, which are crucial for both parasite control and the regulation of inflammation. Additionally, plasma cells play a pivotal role by producing antibodies that specifically target the parasite, thereby assisting in immune-mediated elimination of PF. | [^49,50^](https://www.zotero.org/google-docs/?RbPuvU) |
| Type 1 diabetes  (T1D) | Type 1 diabetes (T1D) is characterized by an autoimmune attack against the insulin-producing beta cells in the pancreas. The immune response in T1D involves the activation of both CD4+ and CD8+ T cells, which erroneously recognize beta-cell antigens as foreign and mount an inflammatory response against them. This inflammatory cascade is further fueled by the secretion of cytokines such as IL-6, IL-17, and IL-21. Additionally, autoantibodies produced by plasma cells target beta-cell antigens, contributing to the ongoing destruction of pancreatic beta-cells. This immune-mediated destruction ultimately leads to insulin deficiency and the clinical manifestations of T1D. | [^51^](https://www.zotero.org/google-docs/?QgGTVJ) |
| Lung transplantation  (LTx) | Following solid organ transplantation, the recipient's immune system may perceive the newly transplanted lung as foreign tissue, triggering an immune response. This response engages various immune cells, including T cells, B cells, and antigen-presenting cells, which collaborate to mount an attack against the transplanted lung tissue. Pro-inflammatory cytokines produced during this process exacerbate inflammation, increasing the risk of rejection of the transplanted lung. Managing this immune response and preventing rejection are critical aspects of post-transplant care in LTx. | [^52^](https://www.zotero.org/google-docs/?YEFNSO) |

**Supplementary Table 3: Innate and adaptive cell response to nine different pathogens at the single-scale infection as predicted by our model.**

| **Infectious Agents Modeled** | **Innate Immune Responses Predicted** | **Adaptive Immune Responses Predicted** | **Supporting References** |
| --- | --- | --- | --- |
| CMV | DCs, NK cells | Th1, CD8+, IgM, IgA, IgG | [^53,54^](https://www.zotero.org/google-docs/?BzR6QN) |
| EBV | DCs, Monocytes, Macrophages, NK cells, Neutrophils | Th1, CD8+, IgM, IgA, IgG | [^55,56^](https://www.zotero.org/google-docs/?5FvqLy) |
| EBOV | DCs, Macrophages, Monocytes | Th1, CD8+, IgM, IgA, IgG | [^57^](https://www.zotero.org/google-docs/?jtAEMs) |
| HIV | DCs, Macrophages | CD8+, IgM, IgA, IgG | [^58^](https://www.zotero.org/google-docs/?nzA5n2) |
| IAV | DCs, NK cells | Th1, CD8+, IgM, IgA, IgG | [^59^](https://www.zotero.org/google-docs/?NmGlg6) |
| SARS-CoV-2 | DCs, Neutrophils, Monocytes, Macrophages | CD8+, IgM, IgA, IgG | [^45,46^](https://www.zotero.org/google-docs/?UT5mFb) |
| MTB | DCs, Macrophages, Neutrophils | Th1, CD8+, IgM, IgA, IgG | [^60^](https://www.zotero.org/google-docs/?6GADBq) |
| Helminth | DCs, Macrophages, | Th2, IgM, IgA | [^48^](https://www.zotero.org/google-docs/?IGSFrA) |
| PF | DCs, Monocytes, Macrophages | Th1, IgM, IgG, IgA | [^61,62^](https://www.zotero.org/google-docs/?H4C6fK) |

**Supplementary Table 4: Experimentally observed behaviors that are reproduced by the model for the nine pathogens.**

| **Disease conditions** | **Supporting references** | **Cytokines** | **Biological scenario,**  **Sentence from original articles** | **Model dynamic behavior** |
| --- | --- | --- | --- | --- |
| CMV | [^63^](https://www.zotero.org/google-docs/?kmaXSd) | IFN-γ and IL-4 | *“Intracellular IFNγ and IL-4 were produced in a dose-dependent manner by T cells in response to CMV only in the seropositive population”* | Agreement |
|  | [^64^](https://www.zotero.org/google-docs/?ljVcco) | TNF-α, IL-6, IL-10, and IL-13,  IL-8 | *“Initial analysis revealed significant increases in the concentrations of the known pro-survival cytokines TNFα, IL-6, and IL-8 along with the chemokine MIP-1α and the immune-regulatory cytokines IL-13 and IL-10”* | Agreement |
|  | [^65^](https://www.zotero.org/google-docs/?sW03Zw) | IL-32 | *“IL-32 levels in sera from actively HCMV-infected patients were significantly higher than those in control groups”* | Disagreement |
|  | - [^66^](https://www.zotero.org/google-docs/?2MXPVi) | IL-1ꞵ | *“As seen in Fig. 1, isolated nonadherent (NAD) monocytes showed a dramatic increase in the steady-state mRNA expression of various monocyte effectors (IL-1b, A20, p105/p50, and IkBa) following a 4-h viral infection (with gradient-purified virus) or incubation with known activating agents (10 ng/ml of PMA or 1 mg/ml of LPS) as compared with the unstimulated control (“NAD”). ”* | Agreement |
| EBV | [^67^](https://www.zotero.org/google-docs/?6DujcI) | IL-13 | *“we found that IL-13 production is induced in B cells early during EBV infection”* | Agreement |
|  | [^68^](https://www.zotero.org/google-docs/?G65UU5) | IL-17 | *“there were significantly more IL-17A-producing cells among CD4+ cells in peripheral blood of CAEBV patients than in that of controls”* | Agreement |
|  | [^69^](https://www.zotero.org/google-docs/?CrzAj3) | IL-2 | *“The IL-2 gene was constantly expressed at higher levels in CD3+ HLA-DR+ cells of patients with chronic active EBV infection”* | Agreement |
|  | [^70^](https://www.zotero.org/google-docs/?QTyXj7) | IL-9 | *“These results suggest that IL-9 induced by EBV-encoded small RNA acts as an autocrine growth factor for EBV-infected T cells.”* | Disagreement |
| EBOV | [^71^](https://www.zotero.org/google-docs/?VJGVr7) | IL-11, IL-17, IL-21, and IL-27  IL-32 | *“The mRNAs encoding several other cytokines, including IL-21, IL-26, IL-32, IL-11, IL-27, and IL-17F, were transiently increased on isolated days.”* | Agreement  Disagreement |
|  | [^72^](https://www.zotero.org/google-docs/?aqpudV) | TGF-ꞵ | *“Upregulation of TGF-β signaling in the kinome data sets correlated with the upregulation of TGF-β secretion from EBOV-infected cells”* | Agreement |
|  | [^73^](https://www.zotero.org/google-docs/?SdCEet) | IL-8 | *“Analysis of supernatants of EBOV-infected PBMCs using a bead-based multiplex assay demonstrated the increased production with both Th1 and Th2 cytokines and CXCL8 chemokine (IL-8), also produced by T cells (*[*58*](https://journals.asm.org/doi/10.1128/mbio.00845-17#core-collateral-B58)*), compared to uninfected PBMCs. “* | Agreement |
| HIV | [^74^](https://www.zotero.org/google-docs/?j8fYSc) | TGF-ꞵ | *“HIV infection resulted in a significant increase of plasma TGF-beta(1) concentration compared to healthy individuals”* | Agreement |
|  | [^75^](https://www.zotero.org/google-docs/?wX55D5) | IL-27 | *“plasma IL-27 level was significantly upregulated in HIV-mono-infected”* | Disagreement |
|  | [^76^](https://www.zotero.org/google-docs/?lBwBKQ) | IL-18 | *“Our main findings were that HIV-1-infected patients had significantly raised IL-18 levels comparing healthy controls, particularly in those with advanced disease”* | Agreement |
|  | [^77^](https://www.zotero.org/google-docs/?RG7Gho) | IL-21 | *“IL-21 is progressively lost during HIV infection, and that its production is associated with control of virus replication in vivo. Another study however reported that HIV infected individuals have greater circulating IL-21 producing CD4 T cells in blood compared to uninfected individuals”* | Partial agreement |
|  | [^78^](https://www.zotero.org/google-docs/?26moVP) | TGF-β | *“It was shown that PBMC from HIV-infected donors overexpress TGFβ, and that this is responsible for some of the defects in T cell function. “* | Agreement |
|  | [^79^](https://www.zotero.org/google-docs/?ucinJV) | IL-29 | *“In HIV-1 infected patients, IL-29 level was increased along with the depletion of CD4⁺ T-cells in peripheral blood, while the elevated IL-29 did not show a significantly negative correlation with viral load.”* | Agreement |
| Influenza A Virus | [^80^](https://www.zotero.org/google-docs/?PK4IEo) | IL-15, IL-12, IL-6 | *“Increased systemic levels of IL-15, IL-12p70, IL-6 constituted a hallmark of critical illness. These mediators are known to promote the development of adaptive responses and also pro-inflammatory ones in other viral infections.”* | Agreement |
|  | [^81^](https://www.zotero.org/google-docs/?zHtPcp) | TGF-β | *“The activity of TGF-β was three times higher in the supernatant from A549 cells treated with PR8 than from those treated with PBS alone”* | Agreement |
|  | [^80^](https://www.zotero.org/google-docs/?XOstAK) | IFN-γ, IL-8, IL-13, and IL-10  IL-9 | *“Increase in IFN-γ IL-8, IL-9, IL-13 and IL-10 in both critical and non critical hospitalized patients compared to mild ones indicates that they constitute hallmarks of severe disease.”* | Agreement  Disagreement |
| SARS-CoV-2 | [^82^](https://www.zotero.org/google-docs/?kZHcOg) | IL-22 | *“IL-22 was detectable neither in plasmas of COVID-19 patients nor in healthy controls”* | Agreement |
|  | [^83^](https://www.zotero.org/google-docs/?rHqHmT) | IL-33 | *patients with COVID-19 had very low IL-33 expression, which was significantly reduced compared to that of control subjects* | Agreement |
|  | [^84^](https://www.zotero.org/google-docs/?YXXyTt) | IL-10, IL-23, and TNF-α | *“Serum levels of IL-23, IL-10, and TNF-α were significantly higher in COVID-19 patients with critical cases compared to mild and severe cases”* | Agreement |
|  | [^85^](https://www.zotero.org/google-docs/?Qrdzqm) | IL-27  IL-32 | *“The levels of IL-27 and IL-32 were significantly higher in COVID-19 patients than healthy subjects (p < 0.0001–0.01)”* | Partial Agreement /  Disagreement |
|  | [^86^](https://www.zotero.org/google-docs/?yOwjrY) | IL-8 | *“Similarly, stimulation of PBMCs with spike glycoprotein S1 (100 ng/mL) for 24 h resulted in ~ 14-fold increase in the production of IL-8.”* | Agreement |
| MTB | [^87^](https://www.zotero.org/google-docs/?f8E4fo) | TGF-β | *“TGFβ levels are increased in blood monocytes isolated from TB patients compared to uninfected individuals”* | Agreement |
|  | [^87^](https://www.zotero.org/google-docs/?P0RM6B) | IL-13 | *“Though not much is known regarding IL-13 in Mtb infection, whole blood mRNA from latently infected children shows increased IL-13 compared to uninfected controls”* | Agreement |
|  | [^88^](https://www.zotero.org/google-docs/?ZeMfQN) | IL-15 | *“High IL-6, high IFN-γ and low IL-15 levels suggest active tuberculosis. Low IL-15 levels indicate an exposure to Mycobacterium tuberculosis.”* | Partial agreement |
| Helminth | [^89^](https://www.zotero.org/google-docs/?Fv6YmT) | IL-1β, IL-6, IL-12, and IL-10 | *“Mantel et al. have demonstrated that the release of RMVs from iRBCs can activate the pro-inﬂammatory cytokines interleukin-6 (IL-6), IL-12, and IL-1b, as well as the anti-inﬂammatory cytokine IL-10, in a dose-dependent manner”* | Agreement |
|  | [^90^](https://www.zotero.org/google-docs/?bkypLq) | TGF-β | *“we identify an H. polygyrus TGF-β mimic (Hp-TGM) that replicates the biological and functional properties of TGF-β, including binding to mammalian TGF-β receptors and inducing mouse and human Foxp3+ Treg cells.”* | Agreement |
|  | [^91^](https://www.zotero.org/google-docs/?JLDStx) | IL-3, IL-4, IL-5, and IL-13  IL-9 | *“The cytokines IL-4, IL-13, IL-3, IL-5, and IL-9 are considered key to helminth induced type 2 immune responses, which together with CD4 T cells, ILC2s, eosinophils, basophils and mast cells form a conserved mechanism that leads to expulsion of helminth species often referred to as the “weep and sweep” response”* | Agreement  Partial Agreement |
| PF | [^92^](https://www.zotero.org/google-docs/?z5Kufg) | IL-6, IL-10, IL-12, IL-13, and IFN-γ | *Data showed that IL-6, IFN-γ, IL-12p70, IL-10, and IL-13 levels were significantly higher in children with symptomatic P. falciparum infection compared to uninfected children* | Agreement |
|  | [^93^](https://www.zotero.org/google-docs/?GJlUuk) | IL-15 | *“IL-15 supports early control and timely resolution of blood-stage malaria through promotion of Th1-dependent innate and adaptive immune responses.”* | Agreement |
|  | [^94^](https://www.zotero.org/google-docs/?lyru4W) | IL-1β, IL-6, GM-CSF, and TGF-β | *IL-1β, IL-6, granulocyte-macrophage colony-stimulating factor, and transforming growth factor (TGF)–β2 were highest in villi cultures.* | Agreement |
|  | [^95^](https://www.zotero.org/google-docs/?jYQNtO) | IL-33 | *“IL-33 serum levels are significantly higher in children with severe Plasmodium falciparum malaria than children without complications or noninfected children.”* | Disagreement |

**Supplementary Table 5: Mechanisms of transplantation phenotypes.**

| **Phenotype** | **Brief Synopsis** | **Citations** |
| --- | --- | --- |
| **Acute Rejection** | Acute rejection in lung transplantation occurs when the immune system of the recipient attacks the donated lung. Damage to lung tissue activates antigen-presenting B cells and IL-33, which in turn activate type 2 innate lymphoid cells, CD8+ T cells, Basophils, and Eosinophils. This initiates a signaling cascade that leads to the activation of CD4+ T cells, Macrophages, and Neutrophils. This signaling pathway leads to the release of Th1- and Th2-response cytokines, IL-6, IL-8, GM-CSF, and varying levels of IL-10, depending on the strength of the response. | [^96^](https://www.zotero.org/google-docs/?UApob5) |
| **Allograft Tolerance** | Allograft tolerance in lung transplantation is the phenomenon in which the recipient’s immune system accepts the transplant. This occurs when there is minimal activity of antigen-presenting cells in the immune system, either due to a lack of activation or to the presence of NK cells, thereby decreasing the population of active antigen-presenting cells. The activity levels of IL-10-producing Tregs are an important factor that correlates with the potential for rejection or tolerance after a lung transplant. | [^96^](https://www.zotero.org/google-docs/?3xk88X) |

**Supplementary Table 6: Model predictions**

* Has an average activity level between 0% and 1% over 100 dose-response simulations

| **Disease Condition** | **Cells Activated** | **Cytokines Released** |
| --- | --- | --- |
| CMV | Basophil_activated, Bcell_APC, Bcell_memory, Bcell_plasma, DC_APC, DC_mature, DC_pDC, Eosinophil_activated, epithelial_CMV, ILC1, ILC2, ILC3, iTreg, Macrophage_M0, Macrophage_M1*, Macrophage_M2, Mastcell_activated, Mastcell_APC_activated, moDC, Monocyte, NeutrophiL_activated, Neutrophil_APC, NK_bright, NK_Dim, Tcell_CD4_activated, Tcell_CD8_cytotoxic, Tgd_activated, Th1, Th17*, Th2, Th9*, Treg* | GM-CSF, IFN-α, IFN-β, IFN-γ, IgA, IgE*, IgG, IgM, IL-10, IL-11, IL-12, IL-13, IL-15, IL-17, IL-18, IL-1α*, IL-1β, IL-21, IL-23, IL-25, IL-27, IL-29, IL-2, IL-3, IL-4, IL-5, IL-6, IL-8, IL-9*, M-CSF, ROS, TGF-β, TNF-α |
| EBV | Basophil_activated, Bcell_APC, Bcell_memory, Bcell_plasma, cDC1, cDC2, DC_APC, DC_mature*, DC_pDC, Eosinophil_activated, epithelial_EBV, ILC1, ILC2, ILC3, iTreg, Macrophage_M0, Macrophage_M1*, Macrophage_M2, Mastcell_activated*, Mastcell_APC_activated*, moDC*, Monocyte, Neutrophil_activated, Neutrophil_APC, NK_bright, NK_Dim, Tcell_CD4_activated*, Tcell_CD8_cytotoxic, Tgd_activated, Th1, Th17, Th2, Th22*, Th9*, Treg* | GM-CSF, IFN-α, IFN-β, IFN-γ, IgA, IgE*, IgG, IgM, IL-10, IL-11, IL-12, IL-13, IL-15, IL-17, IL-18, IL-1α*, IL-1β, IL-21, IL-22*, IL-23, IL-25, IL-27, IL-29, IL-2, IL-3, IL-4, IL-5, IL-6, IL-8, IL-9*, M-CSF, ROS, TGF-β, TNF-α |
| EBOV | Basophil_activated, Bcell_APC, Bcell_memory, Bcell_plasma, cDC2, DC_APC, DC_mature, DC_pDC, Eosinophil_activated, ILC1, ILC2, ILC3, iTreg, Macrophage_M0, Macrophage_M1*, Macrophage_M2*, Mastcell_activated*, MoDC, Monocyte, Neutrophil_activated, Neutrophil_APC, NK_bright, NK_dim, Tcell_CD4_activated*, Tcell_CD8_cytotoxic, Tgd_activated, Th1, Th17*, Th2*, Th9, Treg* | GM-CSF, IFN-α, IFN-β, IFN-γ, IgA, IgE*, IgG, IgM, IL-10, IL-11, IL-12, IL-13, IL-15, IL-17, IL-18, IL-1α*, IL-1β, IL-21, IL-23, IL-25, IL-27, IL-29, IL-2, IL-3, IL-4, IL-5, IL-6, IL-8, IL-9, M-CSF, ROS, TGF-β, TNF-α |
| HIV | Basophil_active, Bcell_APC, Bcell_memory, Bcell_plasma, DC_APC, DC_mature, DC_pDC, Eosinophil_active, epithelial_HIV, ILC1, ILC2*, ILC3, iTreg*, Macrophage_M0, Macrophage_M1*, Macrophage_M2, moDC, Monocyte, Neutrophil_activated, Neutrophil_APC, NK_bright, NK_Dim, Tcell_CD4_activated*, Tcell_CD8_cytotoxic, Tgd_activated, Th1*, Th17*, Th2*, Th9*, Treg | GM-CSF, IFN-α, IFN-β, IFN-γ, IgA, IgE*, IgG, IgM, IL-10, IL-11, IL-12, IL-13, IL-15, IL-17, IL-18, IL-1α*, IL-1β, IL-21, IL-23, IL-25, IL-27, IL-29, IL-2, IL-3, IL-4, IL-5, IL-6, IL-8, IL-9*, M-CSF, ROS, TGF-β, TNF-α |
| IAV | Basophil_active, Bcell_APC, Bcell_memory, Bcell_plasma, cDC1, DC_APC, DC_mature, DC_pDC, Eosinophil_active, epithelial_IAV, ILC1, ILC2, ILC3, iTreg, Macrophage_M0, Macrophage_M1, Macrophage_M2, Mastcell_activated, Mastcell_APC_activated, moDC, Monocyte, Neutrophil_activated, Neutrophil_APC, NK_bright, NK_dim, Tcell_CD4_activated, Tcell_CD8_activated, Tgd_activated, Th1, Th17, Th2, Th22, Th9, Treg | GM-CSF, IFN-α, IFN-β, IFN-γ, IgA, IgE, IgG, IgM, IL-10, IL-11, IL-12, IL-13, IL-15, IL-17, IL-18, IL-1α, IL-1β, IL-21, IL-22, IL-23, IL-25, IL-27, IL-29, IL-2, IL-32, IL-33, IL-35, IL-3, IL-4, IL-5, IL-6, IL-8, IL-9, M-CSF, ROS, TGF-β, TNF-α |
| SARS-CoV-2 | Basophil_activated, Bcell_APC, Bcell_memory, Bcell_plasma, DC_APC, DC_mature, DC_pDC, Eosinophil_activated, epithalial_damaged_cells, epithelial_SARSCoV2, ILC1, ILC2, ILC3, iTreg, Macrophage_M0, Macrophage_M1*, Macrophage_M2, Mastcell_activated*, moDC, Monocyte, Neutrophil_activated, Neutrophil_APC, NK_bright, NK_Dim, Tcell_CD4_activated, Tcell_CD8_cytotoxic, Tgd_activated, Th1, Th17*, Th2, Th22, Th9, Treg* | GM-CSF, IFN-α, IFN-β, IFN-γ, IgA, IgE*, IgG, IgM, IL-10, IL-11, IL-12, IL-13, IL-15, IL-17, IL-18, IL-1α*, IL-1β, IL-21, IL-22, IL-23, IL-25, IL-27, IL-29, IL-2, IL-33, IL-3, IL-4, IL-5, IL-6, IL-8, IL-9, M-CSF, ROS, TGF-β, TNF-α |
| MTB | Basophil_activated, Bcell_APC, Bcell_memory, Bcell_plasma, cDC1, cDC2, DC_APC, DC_mature, DC_pDC, Eosinophil_active, epithelial_MTB*, ILC1, ILC2, ILC3, iTreg, Macrophage_M0, Macrophage_M1*, Macrophage_M2, Mastcell_activated, Mastcell_APC_activated, MoDC, Monocyte, Neutrophil_activated, Neutrophil_APC, NK_bright, NK_Dim, Tcell_CD4_activated, Tcell_CD8_activated, Tgd_activated, Th1, Th17, Th2, Th22, Th9, Treg | GM-CSF, IFN-α, IFN-β, IFN-γ, IgA, IgE, IgG, IgM, IL-10, IL-11, IL-12, IL-13, IL-15, IL-17, IL-18, IL-1α, IL-1β, IL-21, IL-22, IL-23, IL-25, IL-27, IL-29, IL-2, IL-35, IL-3, IL-4, IL-5, IL-6, IL-8, IL-9, M-CSF, ROS, TGF-β, TNF-α |
| Helminth | Basophil_activated, Bcell_APC, Bcell_memory, Bcell_plasma, cDC2, DC_APC, DC_mature, Eosinophil_activated, epithelial_Helminth, ILC1, ILC2*, ILC3, iTreg, Macrophage_M0, Macrophage_M1*, Macrophage_M2, Mastcell_activated, Mastcell_APC_activated, MoDC, Monocyte, Neutrophil_activated, Neutrophil_APC, NK_bright, NK_Dim, Tcell_CD4_activated*, Tcell_CD8_cytotoxic, Tgd_activated, Th1, Th17*, Th2, Th9, Treg | GM-CSF, IFN-α, IFN-β, IFN-γ, IgA, IgE, IgG, IgM, IL-10, IL-11, IL-12, IL-13, IL-15, IL-17, IL-18, IL-1α*, IL-1β, IL-21, IL-23, IL-25, IL-27, IL-29, IL-2, IL-33, IL-3, IL-4, IL-5, IL-6, IL-8*, IL-9, M-CSF, ROS, TGF-β, TNF-α |
| PF | Basaophil_activated, Bcell_APC, Bcell_memory, Bcell_plasma, cDC2, DC_APC, DC_mature*, Eosinophil_activated, ILC1, ILC2, ILC3, iTreg, Macrophage_M0, Macrophage_M1*, Macrophage_M2, Mastcell_activated, Mastcell_APC, activated, moDC, Monocyte, Neutrophil_activated, Neutrophil_APC, NK_bright, NK_Dim, RBC_PF, Tcell_CD4_activated, Tcell_CD8_cytotoxic, Tgd_activated, Th1, Th2, Th9, Treg* | GM-CSF, IFN-α, IFN-β, IFN-γ, IgA, IgE, IgG, IgM, IL-10, IL-11, IL-12, IL-13, IL-15, IL-17, IL-18, IL-1α*, IL-1β, IL-21, IL-23, IL-25, IL-27, IL-29, IL-2, IL-3, IL-4, IL-5, IL-6, IL-8, IL-9, M-CSF, ROS, TGF-β, TNF-α |
| Diabetes | Basophil_activated, Bcell_APC, Bcell_memory, Bcell_plasma, DC_APC, DC_mature, Eosinophil_activated*, ILC1, ILC2, ILC3, iTreg, Macrophage_M0, Macrophage_M1*, Macrophage_M2*, Mastcell_activated, MoDC, Monocyte, Neutrophil_activated, Neutrophil_APC, NK_bright, NK_Dim, pancreatic_beta_damaged_cells, Tcell_CD4_activated*, Tcell_CD8_cytotoxic, Tgd_activated, Th1, Th17*, Th2*, Th9* | GM-CSF, IFN-α, IFN-β, IFN-γ, IgA, IgG, IgM, IL-10, IL-11, IL-12, IL-13, IL-15, IL-17, IL-18, IL-1α*, IL-1β, IL-21, IL-23, IL-25, IL-27, IL-29, IL-2, IL-3, IL-4, IL-5, IL-6, IL-8*, IL-9*, M-CSF, ROS, TGF-β, TNF-α |
| LTx | Basophil_activated, Bcell_APC, Bcell_memory, Bcell_plasma, DC_APC, DC_mature, Eosinophil_activated, ILC1, ILC2, ILC3, iTreg, Macrophage_M0, Macrophage_M2, Mastcell_activated, Mastcell_APC_activated, moDC, Monocyte, Neutrophil_activated, Neutrophil_APC, NK_bright, NK_Dim, Tcell_CD4_activated, Tcell_CD8_cytotoxic, Tgd_activated, Th1, Th17*, Th2, Th9*, Treg | GM-CSF, IFN-α, IFN-β, IFN-γ, IgA, IgE, IgG, IgM, IL-10, IL-11, IL-12, IL-13, IL-15, IL-17, IL-18, IL-1β, IL-21, IL-23, IL-25, IL-27, IL-29, IL-2, IL-33, IL-3, IL-4, IL-5, IL-6, IL-8, IL-9*, M-CSF, ROS, TGF-β, TNF-α |
| MTB + HIV Coinfection | Basophil_activated, Bcell_APC, Bcell_memory, Bcell_plasma, cDC1, cDC2, DC_APC, DC_mature, DC_pDC, Eosinophil_activated, epithelial_HIV, epithelial_MTB*, ILC1, ILC2*, ILC3, iTreg*, Macrophage_M0, Macrophage_M1, Macrophage_M2, Mastcell_activated, Mastcell_APC_activated*, moDC, Monocyte, Neutrophil_activated, Neutrophil_APC, NK_bright, NK_Dim, Tcell_CD4_activated*, Tcell_CD8_cytotoxic, Tgd_activated, Th1*, Th17*, Th2, Th22*, Th9*, Treg* | GM-CSF, IFN-α, IFN-β, IFN-γ, IgA, IgE*, IgG, IgM, IL-10, IL-11, IL-12, IL-13, IL-15, IL-17, IL-18, IL-1α, IL-1β, IL-21, IL-22*, IL-23, IL-25, IL-27, IL-29, IL-2, IL-35, IL-3, IL-4, IL-5, IL-6, IL-8, IL-9*, M-CSF, ROS, TGF-β, TNF-α |
| MTB + Helminth Coinfection | Basophil_activated, Bcell_APC, Bcell_memory, Bcell_plasma, cDC1, cDC2, DC_APC, DC_mature, DC_pDC, Eosinophil_activated, epithelial_Helminth, epithelial_MTB*, ILC1, ILC2*, ILC3, iTreg, Macrophage_M0, Macrophage_M1*, Macrophage_M2, Mastcell_activated, Mastcell_APC_activated, moDC, Monocyte, Neutrophil_activated, Neutrophil_APC, NK_bright, NK_Dim, Tcell_CD4_activated*, Tcell_CD8_cytotoxic, Tgd_activated, Th1, Th17, Th2, Th22*, Th9, Treg | GM-CSF, IFN-α, IFN-β, IFN-γ, IgA, IgE, IgG, IgM, IL-10, IL-11, IL-12, IL-13, IL-15, IL-17, IL-18, IL-1α, IL-1β, IL-21, IL-22, IL-23, IL-25, IL-27, IL-29, IL-2, IL-33, IL-35, IL-3, IL-4, IL-5, IL-6, IL-8, IL-9, M-CSF, ROS, TGF-β, TNF-α |
| SARS-CoV-2 + EBV Coinfection | Basophil_activated, Bcell_APC, Bcell_memory, Bcell_plasma, cDC1, cDC2, DC_APC, DC_mature, DC_pDC, Eosinophil_active, epithelial_damaged_cells, epithelial_EBV, epithelial_SARSCoV2, ILC1, ILC2, ILC3, iTreg, Macrophage_M0, Macrophage_M1*, Macrophage_M2, moDC, Monocyte, Neutrophil_activated, Neutrophil_APC, NK_bright, NK_Dim, Tcell_CD4_activated*, Tcell_CD8_cytotoxic, Tgd_activated, Th1, Th17*, Th2*, Th22, Th9, Treg* | GM-CSF, IFN-α, IFN-β, IFN-γ, IgA, IgE*, IgG, IgM, IL-10, IL-11, IL-12, IL-13, IL-15, IL-17, IL-8, IL-1α, IL-1β, IL-21, IL-22, IL-23, IL-25, IL-27, IL-29, IL-2, IL-33, IL-3, IL-4, IL-5, IL-6, IL-8, IL-9, M-CSF, ROS, TGF-β, TNF-α |
| PF + Helminth Coinfection | Basophil_activated, Bcell_APC, Bcell_memory, Bcell_plasma, cDC2, DC_APC, DC_mature, Eosinophil_activated, epithelial_Helminth, ILC1, ILC2*, ILC3, iTreg, Macrophage_M0, Macrophage_M1, Macrophage_M2, Mastcell_activated, Mastcell_APC_activated, moDC, Monocyte, Neutrophil_activated, Neutrophil_APC, NK_bright, NK_Dim, RBC_PF, Tcell_CD4_activated*, Tcell_CD8_cytotoxic, Tgd_activated, Th1, Th17*, Th2, Th9, Treg | GM-CSF, IFN-α, IFN-β, IFN-γ, IgA, IgE, IgG, IgM, IL-11, IL-12, IL-13, IL-15, IL-17, IL-18, IL-1α, IL-1β, IL-21, IL-23, IL-25, IL-27, IL-29, IL-2, IL-33, IL-3, IL-4, IL-5, IL-6, IL-8, IL-9, M-CSF, ROS, TGF-β, TNF-α |

**Supplementary Table 7: Coinfection mechanisms**

| **Disease Condition #1** | **Disease Condition #2** | **Mechanism** | **Citations** |
| --- | --- | --- | --- |
| MTB | HIV | Coinfection with MTB and HIV induces the activation of the B cells, Neutrophils, NK cells, and CD8+ T cells, the activity of CD4+ T cells, Th1 and Th2 cells, and macrophages is suppressed, unlike during monoinfection. The pro-inflammatory cytokines TNF-α, IFN-γ, and IL-6 remain active in the coinfection because they are produced by responding cells. | [^97,98^](https://www.zotero.org/google-docs/?QtRKLV) |
| MTB | Helminth | In an MTB and Helminth coinfection, a signaling cascade is triggered through epithelial cells to antigen-presenting cells and type 2 cytokine-producing cells, which then activate CD4+ T cells and their effector cell populations. This coinfection results in a combination of type 1, type 2, and regulatory cytokines, with the prominence of each type influenced by the stage of each infection. | [^99^](https://www.zotero.org/google-docs/?0vZTLs) |
| SARS-CoV-2 | EBV | In SARS-CoV-2 and EBV coinfections, the activity of SARS-CoV-2 causes the reactivation of latent EBV through the suppression of CD8+ T cell activity. This results in the release of high levels of Th1, Th2, and Th17 cytokines. | [^100,101^](https://www.zotero.org/google-docs/?FMrWsf) |
| PF | Helminth | Coinfection with PF and Helminth begins with infection of red blood cells and epithelial cells, which activate antigen-presenting cells to stimulate T cell activity, and macrophages and neutrophils to manage parasitemia. This cascade induces an immune response characterized by the activity of Th1, Th2, and Treg cytokines, with the presence of these cytokines dependent on the stage of infection. | [^102^](https://www.zotero.org/google-docs/?pReKBf) |

**References:**

[1.](https://www.zotero.org/google-docs/?UYei3x) [Heijink, I. H. *et al.* Epithelial cell dysfunction, a major driver of asthma development. *Allergy* **75**, 1902–1917 (2020).](https://www.zotero.org/google-docs/?UYei3x)

[2.](https://www.zotero.org/google-docs/?UYei3x) [Barbalato, L. & Pillarisetty, L. S. *Histology, Red Blood Cell*. (StatPearls Publishing, 2024).](https://www.zotero.org/google-docs/?UYei3x)

[3.](https://www.zotero.org/google-docs/?UYei3x) [Siracusa, M. C., Kim, B. S., Spergel, J. M. & Artis, D. Basophils and allergic inflammation. *J. Allergy Clin. Immunol.* **132**, 789–801 (2013).](https://www.zotero.org/google-docs/?UYei3x)

[4.](https://www.zotero.org/google-docs/?UYei3x) [Sokol, C. L. *et al.* Basophils Function as Antigen Presenting Cells for an Allergen-Induced TH2 Response. *Nat. Immunol.* **10**, 713–720 (2009).](https://www.zotero.org/google-docs/?UYei3x)

[5.](https://www.zotero.org/google-docs/?UYei3x) [Aghamiri, S. S., Puniya, B. L., Amin, R. & Helikar, T. A multiscale mechanistic model of human dendritic cells for in-silico investigation of immune responses and novel therapeutics discovery. *Front. Immunol.* **14**, 1112985 (2023).](https://www.zotero.org/google-docs/?UYei3x)

[6.](https://www.zotero.org/google-docs/?UYei3x) [Amon, L., Lehmann, C. H. K., Heger, L., Heidkamp, G. F. & Dudziak, D. The ontogenetic path of human dendritic cells. *Mol. Immunol.* **120**, 122–129 (2020).](https://www.zotero.org/google-docs/?UYei3x)

[7.](https://www.zotero.org/google-docs/?UYei3x) [Liu, K. Dendritic Cells. *Encycl. Cell Biol.* **3**, 741–749 (2016).](https://www.zotero.org/google-docs/?UYei3x)

[8.](https://www.zotero.org/google-docs/?UYei3x) [Elkord, E., Williams, P. E., Kynaston, H. & Rowbottom, A. W. Human monocyte isolation methods influence cytokine production from in vitro generated dendritic cells. *Immunology* **114**, 204–212 (2005).](https://www.zotero.org/google-docs/?UYei3x)

[9.](https://www.zotero.org/google-docs/?UYei3x) [Segura, E. Human dendritic cell subsets: An updated view of their ontogeny and functional specialization. *Eur. J. Immunol.* **52**, 1759–1767 (2022).](https://www.zotero.org/google-docs/?UYei3x)

[10.](https://www.zotero.org/google-docs/?UYei3x) [Heger, L. *et al.* XCR1 expression distinguishes human conventional dendritic cell type 1 with full effector functions from their immediate precursors. *Proc. Natl. Acad. Sci.* **120**, e2300343120 (2023).](https://www.zotero.org/google-docs/?UYei3x)

[11.](https://www.zotero.org/google-docs/?UYei3x) [Chistiakov, D. A., Orekhov, A. N., Sobenin, I. A. & Bobryshev, Y. V. Plasmacytoid dendritic cells: development, functions, and role in atherosclerotic inflammation. *Front. Physiol.* **5**, 279 (2014).](https://www.zotero.org/google-docs/?UYei3x)

[12.](https://www.zotero.org/google-docs/?UYei3x) [Geginat, J. *et al.* Immunity to Pathogens Taught by Specialized Human Dendritic Cell Subsets. *Front. Immunol.* **6**, 527 (2015).](https://www.zotero.org/google-docs/?UYei3x)

[13.](https://www.zotero.org/google-docs/?UYei3x) [Kita, H. Eosinophils: Multifaceted Biologic Properties and Roles in Health and Disease. *Immunol. Rev.* **242**, 161–177 (2011).](https://www.zotero.org/google-docs/?UYei3x)

[14.](https://www.zotero.org/google-docs/?UYei3x) [Jin, J., Sunusi, S. & Lu, H. Group 2 innate lymphoid cells (ILC2s) are important in typical type 2 immune-mediated diseases and an essential therapeutic target. *J. Int. Med. Res.* **50**, 03000605211053156 (2022).](https://www.zotero.org/google-docs/?UYei3x)

[15.](https://www.zotero.org/google-docs/?UYei3x) [Taggenbrock, R. L. R. E. & van Gisbergen, K. P. J. M. ILC1: Development, maturation, and transcriptional regulation. *Eur. J. Immunol.* **53**, e2149435 (2023).](https://www.zotero.org/google-docs/?UYei3x)

[16.](https://www.zotero.org/google-docs/?UYei3x) [Herbert, D. R., Douglas, B. & Zullo, K. Group 2 Innate Lymphoid Cells (ILC2): Type 2 Immunity and Helminth Immunity. *Int. J. Mol. Sci.* **20**, 2276 (2019).](https://www.zotero.org/google-docs/?UYei3x)

[17.](https://www.zotero.org/google-docs/?UYei3x) [Valle-Noguera, A., Ochoa-Ramos, A., Gomez-Sánchez, M. J. & Cruz-Adalia, A. Type 3 Innate Lymphoid Cells as Regulators of the Host-Pathogen Interaction. *Front. Immunol.* **12**, 748851 (2021).](https://www.zotero.org/google-docs/?UYei3x)

[18.](https://www.zotero.org/google-docs/?UYei3x) [Ross, E. A., Devitt, A. & Johnson, J. R. Macrophages: The Good, the Bad, and the Gluttony. *Front. Immunol.* **12**, 708186 (2021).](https://www.zotero.org/google-docs/?UYei3x)

[19.](https://www.zotero.org/google-docs/?UYei3x) [Atri, C., Guerfali, F. Z. & Laouini, D. Role of Human Macrophage Polarization in Inflammation during Infectious Diseases. *Int. J. Mol. Sci.* **19**, 1801 (2018).](https://www.zotero.org/google-docs/?UYei3x)

[20.](https://www.zotero.org/google-docs/?UYei3x) [Krystel-Whittemore, M., Dileepan, K. N. & Wood, J. G. Mast Cell: A Multi-Functional Master Cell. *Front. Immunol.* **6**, 620 (2016).](https://www.zotero.org/google-docs/?UYei3x)

[21.](https://www.zotero.org/google-docs/?UYei3x) [Serbina, N. V., Jia, T., Hohl, T. M. & Pamer, E. G. Monocyte-Mediated Defense Against Microbial Pathogens. *Annu. Rev. Immunol.* **26**, 421–452 (2008).](https://www.zotero.org/google-docs/?UYei3x)

[22.](https://www.zotero.org/google-docs/?UYei3x) [Selders, G. S., Fetz, A. E., Radic, M. Z. & Bowlin, G. L. An overview of the role of neutrophils in innate immunity, inflammation and host-biomaterial integration. *Regen. Biomater.* **4**, 55–68 (2017).](https://www.zotero.org/google-docs/?UYei3x)

[23.](https://www.zotero.org/google-docs/?UYei3x) [Abel, A. M., Yang, C., Thakar, M. S. & Malarkannan, S. Natural Killer Cells: Development, Maturation, and Clinical Utilization. *Front. Immunol.* **9**, 1869 (2018).](https://www.zotero.org/google-docs/?UYei3x)

[24.](https://www.zotero.org/google-docs/?UYei3x) [Björkström, N. K., Strunz, B. & Ljunggren, H.-G. Natural killer cells in antiviral immunity. *Nat. Rev. Immunol.* **22**, 112–123 (2022).](https://www.zotero.org/google-docs/?UYei3x)

[25.](https://www.zotero.org/google-docs/?UYei3x) [Janeway, C. A., Travers, P., Walport, M. & Shlomchik, M. J. B-cell activation by armed helper T cells. in *Immunobiology: The Immune System in Health and Disease. 5th edition* 402–426 (Garland Science, 2001).](https://www.zotero.org/google-docs/?UYei3x)

[26.](https://www.zotero.org/google-docs/?UYei3x) [Zhu, J., Yamane, H. & Paul, W. E. Differentiation of effector CD4 T cell populations (*). *Annu. Rev. Immunol.* **28**, 445–489 (2010).](https://www.zotero.org/google-docs/?UYei3x)

[27.](https://www.zotero.org/google-docs/?UYei3x) [Luckheeram, R. V., Zhou, R., Verma, A. D. & Xia, B. CD4+T Cells: Differentiation and Functions. *J. Immunol. Res.* **2012**, 925135 (2012).](https://www.zotero.org/google-docs/?UYei3x)

[28.](https://www.zotero.org/google-docs/?UYei3x) [Puniya, B. L. *et al.* A Mechanistic Computational Model Reveals That Plasticity of CD4+ T Cell Differentiation Is a Function of Cytokine Composition and Dosage. *Front. Physiol.* **9**, 878 (2018).](https://www.zotero.org/google-docs/?UYei3x)

[29.](https://www.zotero.org/google-docs/?UYei3x) [Zhu, L. *et al.* Single-Cell Sequencing of Peripheral Mononuclear Cells Reveals Distinct Immune Response Landscapes of COVID-19 and Influenza Patients. *Immunity* **53**, 685-696.e3 (2020).](https://www.zotero.org/google-docs/?UYei3x)

[30.](https://www.zotero.org/google-docs/?UYei3x) [Chen, J. *et al.* T Helper 9 Cells: A New Player in Immune-Related Diseases. *DNA Cell Biol.* **38**, 1040–1047 (2019).](https://www.zotero.org/google-docs/?UYei3x)

[31.](https://www.zotero.org/google-docs/?UYei3x) [Harrington, L. E. *et al.* Interleukin 17-producing CD4+ effector T cells develop via a lineage distinct from the T helper type 1 and 2 lineages. *Nat. Immunol.* **6**, 1123–1132 (2005).](https://www.zotero.org/google-docs/?UYei3x)

[32.](https://www.zotero.org/google-docs/?UYei3x) [Zhang, K., Chen, L., Zhu, C., Zhang, M. & Liang, C. Current Knowledge of Th22 Cell and IL-22 Functions in Infectious Diseases. *Pathogens* **12**, 176 (2023).](https://www.zotero.org/google-docs/?UYei3x)

[33.](https://www.zotero.org/google-docs/?UYei3x) [Zhang, N. & Bevan, M. J. CD8+ T Cells: Foot Soldiers of the Immune System. *Immunity* **35**, 161–168 (2011).](https://www.zotero.org/google-docs/?UYei3x)

[34.](https://www.zotero.org/google-docs/?UYei3x) [Janeway, C. A., Travers, P., Walport, M. & Shlomchik, M. J. T cell-mediated cytotoxicity. in *Immunobiology: The Immune System in Health and Disease. 5th edition* 383–390 (Garland Science, 2001).](https://www.zotero.org/google-docs/?UYei3x)

[35.](https://www.zotero.org/google-docs/?UYei3x) [Born, W. K., Reardon, C. L. & O’Brien, R. L. The function of gammadelta T cells in innate immunity. *Curr. Opin. Immunol.* **18**, 31–38 (2006).](https://www.zotero.org/google-docs/?UYei3x)

[36.](https://www.zotero.org/google-docs/?UYei3x) [Griffiths, P. & Reeves, M. Pathogenesis of human cytomegalovirus in the immunocompromised host. *Nat. Rev. Microbiol.* **19**, 759–773 (2021).](https://www.zotero.org/google-docs/?UYei3x)

[37.](https://www.zotero.org/google-docs/?UYei3x) [Albanese, M., Tagawa, T. & Hammerschmidt, W. Strategies of Epstein-Barr virus to evade innate antiviral immunity of its human host. *Front. Microbiol.* **13**, 955603 (2022).](https://www.zotero.org/google-docs/?UYei3x)

[38.](https://www.zotero.org/google-docs/?UYei3x) [Münz, C. Epstein-Barr Virus-Specific Immune Control by Innate Lymphocytes. *Front. Immunol.* **8**, 1658 (2017).](https://www.zotero.org/google-docs/?UYei3x)

[39.](https://www.zotero.org/google-docs/?UYei3x) [Baize, S. *et al.* Inflammatory responses in Ebola virus-infected patients. *Clin. Exp. Immunol.* **128**, 163–168 (2002).](https://www.zotero.org/google-docs/?UYei3x)

[40.](https://www.zotero.org/google-docs/?UYei3x) [Olukitibi, T. A., Ao, Z., Mahmoudi, M., Kobinger, G. A. & Yao, X. Dendritic Cells/Macrophages-Targeting Feature of Ebola Glycoprotein and its Potential as Immunological Facilitator for Antiviral Vaccine Approach. *Microorganisms* **7**, 402 (2019).](https://www.zotero.org/google-docs/?UYei3x)

[41.](https://www.zotero.org/google-docs/?UYei3x) [Carrington, M. & Alter, G. Innate Immune Control of HIV. *Cold Spring Harb. Perspect. Med.* **2**, a007070 (2012).](https://www.zotero.org/google-docs/?UYei3x)

[42.](https://www.zotero.org/google-docs/?UYei3x) [Chen, X. *et al.* Host Immune Response to Influenza A Virus Infection. *Front. Immunol.* **9**, 320 (2018).](https://www.zotero.org/google-docs/?UYei3x)

[43.](https://www.zotero.org/google-docs/?UYei3x) [Zhai, W., Wu, F., Zhang, Y., Fu, Y. & Liu, Z. The Immune Escape Mechanisms of Mycobacterium Tuberculosis. *Int. J. Mol. Sci.* **20**, 340 (2019).](https://www.zotero.org/google-docs/?UYei3x)

[44.](https://www.zotero.org/google-docs/?UYei3x) [Caccamo, N. *et al.* Human CD8 T lymphocytes recognize Mycobacterium tuberculosis antigens presented by HLA-E during active tuberculosis and express type 2 cytokines. *Eur. J. Immunol.* **45**, 1069–1081 (2015).](https://www.zotero.org/google-docs/?UYei3x)

[45.](https://www.zotero.org/google-docs/?UYei3x) [Zhou, X. & Ye, Q. Cellular Immune Response to COVID-19 and Potential Immune Modulators. *Front. Immunol.* **12**, 646333 (2021).](https://www.zotero.org/google-docs/?UYei3x)

[46.](https://www.zotero.org/google-docs/?UYei3x) [Al-Attiyah, R. *et al.* Immune Cells Profiles In The Peripheral Blood Of Patients With Moderate To Severe COVID-19 And Healthy Subjects With and Without Vaccination With The Pfizer-BioNTech mRNA Vaccine. *Front. Immunol.* **13**, 851765 (2022).](https://www.zotero.org/google-docs/?UYei3x)

[47.](https://www.zotero.org/google-docs/?UYei3x) [Babu, S. & Nutman, T. B. 31 - Immune Responses to Helminth Infection. in *Clinical Immunology (Fifth Edition)* (eds Rich, R. R. et al.) 437-447.e1 (Elsevier, 2019).](https://www.zotero.org/google-docs/?UYei3x)

[48.](https://www.zotero.org/google-docs/?UYei3x) [Motran, C. C. *et al.* Helminth Infections: Recognition and Modulation of the Immune Response by Innate Immune Cells. *Front. Immunol.* **9**, 664 (2018).](https://www.zotero.org/google-docs/?UYei3x)

[49.](https://www.zotero.org/google-docs/?UYei3x) [Bucşan, A. N. & Williamson, K. C. Setting the stage: The initial immune response to blood-stage parasites. *Virulence* **11**, 88–103 (2020).](https://www.zotero.org/google-docs/?UYei3x)

[50.](https://www.zotero.org/google-docs/?UYei3x) [Stevenson, M. M. & Riley, E. M. Innate immunity to malaria. *Nat. Rev. Immunol.* **4**, 169–180 (2004).](https://www.zotero.org/google-docs/?UYei3x)

[51.](https://www.zotero.org/google-docs/?UYei3x) [Lu, J., Liu, J., Li, L., Lan, Y. & Liang, Y. Cytokines in type 1 diabetes: mechanisms of action and immunotherapeutic targets. *Clin. Transl. Immunol.* **9**, e1122 (2020).](https://www.zotero.org/google-docs/?UYei3x)

[52.](https://www.zotero.org/google-docs/?UYei3x) [Corris, P. A. & Kirby, J. A. A role for cytokine measurement in therapeutic monitoring of immunosuppressive drugs following lung transplantation. *Clin. Exp. Immunol.* **139**, 176–178 (2005).](https://www.zotero.org/google-docs/?UYei3x)

[53.](https://www.zotero.org/google-docs/?UYei3x) [Levy, E. & Sarov, I. Determination of IgA antibodies to human cytomegalovirus by enzyme-linked immunosorbent assay (ELISA). *J. Med. Virol.* **6**, 249–257 (1980).](https://www.zotero.org/google-docs/?UYei3x)

[54.](https://www.zotero.org/google-docs/?UYei3x) [van de Berg, P. J. *et al.* Human cytomegalovirus induces systemic immune activation characterized by a type 1 cytokine signature. *J. Infect. Dis.* **202**, 690–699 (2010).](https://www.zotero.org/google-docs/?UYei3x)

[55.](https://www.zotero.org/google-docs/?UYei3x) [Chijioke, O., Azzi, T., Nadal, D. & Münz, C. Innate immune responses against Epstein Barr virus infection. *J. Leukoc. Biol.* **94**, 1185–1190 (2013).](https://www.zotero.org/google-docs/?UYei3x)

[56.](https://www.zotero.org/google-docs/?UYei3x) [Martorelli, D. *et al.* Exploiting the interplay between innate and adaptive immunity to improve immunotherapeutic strategies for Epstein-Barr-virus-driven disorders. *Clin. Dev. Immunol.* **2012**, 931952 (2012).](https://www.zotero.org/google-docs/?UYei3x)

[57.](https://www.zotero.org/google-docs/?UYei3x) [Leroy, E. M., Baize, S., Debre, P., Lansoud-Soukate, J. & Mavoungou, E. Early immune responses accompanying human asymptomatic Ebola infections. *Clin. Exp. Immunol.* **124**, 453–460 (2001).](https://www.zotero.org/google-docs/?UYei3x)

[58.](https://www.zotero.org/google-docs/?UYei3x) [Banks, H. T., Davidian, M., Hu, S., Kepler, G. M. & Rosenberg, E. S. Modelling HIV immune response and validation with clinical data. *J. Biol. Dyn.* **2**, 357–385 (2008).](https://www.zotero.org/google-docs/?UYei3x)

[59.](https://www.zotero.org/google-docs/?UYei3x) [Ada, G. L. & Jones, P. D. The Immune Response to Influenza Infection. in *Current Topics in Microbiology and Immunology* (eds Clarke, A. et al.) 1–54 (Springer Nature, 1986).](https://www.zotero.org/google-docs/?UYei3x)

[60.](https://www.zotero.org/google-docs/?UYei3x) [O’Garra, A. *et al.* The immune response in tuberculosis. *Annu. Rev. Immunol.* **31**, 475–527 (2013).](https://www.zotero.org/google-docs/?UYei3x)

[61.](https://www.zotero.org/google-docs/?UYei3x) [Yap, X. Z., Lundie, R. J., Beeson, J. G. & O’Keeffe, M. Dendritic Cell Responses and Function in Malaria. *Front. Immunol.* **10**, 357 (2019).](https://www.zotero.org/google-docs/?UYei3x)

[62.](https://www.zotero.org/google-docs/?UYei3x) [Ozarslan, N., Robinson, J. F. & Gaw, S. L. Circulating Monocytes, Tissue Macrophages, and Malaria. *J. Trop. Med.* **2019**, 3720838 (2019).](https://www.zotero.org/google-docs/?UYei3x)

[63.](https://www.zotero.org/google-docs/?UYei3x) [Kallas, E. G. *et al.* Cytomegalovirus-specific IFNgamma and IL-4 are produced by antigen expanded human blood lymphocytes from seropositive volunteers. *Immunol. Lett.* **64**, 63–69 (1998).](https://www.zotero.org/google-docs/?UYei3x)

[64.](https://www.zotero.org/google-docs/?UYei3x) [Pocock, J. M. *et al.* Human Cytomegalovirus Delays Neutrophil Apoptosis and Stimulates the Release of a Prosurvival Secretome. *Front. Immunol.* **8**, 1185 (2017).](https://www.zotero.org/google-docs/?UYei3x)

[65.](https://www.zotero.org/google-docs/?UYei3x) [Huang, Y. *et al.* The expression of interleukin-32 is activated by human cytomegalovirus infection and down regulated by hcmv-miR-UL112-1. *Virol. J.* **10**, 51 (2013).](https://www.zotero.org/google-docs/?UYei3x)

[66.](https://www.zotero.org/google-docs/?UYei3x) [Yurochko, A. D. & Huang, E.-S. Human Cytomegalovirus Binding to Human Monocytes Induces Immunoregulatory Gene Expression1. *J. Immunol.* **162**, 4806–4816 (1999).](https://www.zotero.org/google-docs/?UYei3x)

[67.](https://www.zotero.org/google-docs/?UYei3x) [Tsai, S. C. *et al.* EBV Zta protein induces the expression of interleukin-13, promoting the proliferation of EBV-infected B cells and lymphoblastoid cell lines. *Blood* **114**, 109–118 (2009).](https://www.zotero.org/google-docs/?UYei3x)

[68.](https://www.zotero.org/google-docs/?UYei3x) [Ohta, R., Imai, M., Kawada, J., Kimura, H. & Ito, Y. Interleukin-17A-producing T lymphocytes in chronic active Epstein-Barr virus infection. *Microbiol. Immunol.* **57**, 139–144 (2013).](https://www.zotero.org/google-docs/?UYei3x)

[69.](https://www.zotero.org/google-docs/?UYei3x) [Ohga, S. *et al.* Epstein-Barr virus (EBV) load and cytokine gene expression in activated T cells of chronic active EBV infection. *J. Infect. Dis.* **183**, 1–7 (2001).](https://www.zotero.org/google-docs/?UYei3x)

[70.](https://www.zotero.org/google-docs/?UYei3x) [Yang, L., Aozasa, K., Oshimi, K. & Takada, K. Epstein-Barr virus (EBV)-encoded RNA promotes growth of EBV-infected T cells through interleukin-9 induction. *Cancer Res.* **64**, 5332–5337 (2004).](https://www.zotero.org/google-docs/?UYei3x)

[71.](https://www.zotero.org/google-docs/?UYei3x) [Kash, J. C. *et al.* Longitudinal peripheral blood transcriptional analysis of a patient with severe Ebola virus disease. *Sci. Transl. Med.* **9**, eaai9321 (2017).](https://www.zotero.org/google-docs/?UYei3x)

[72.](https://www.zotero.org/google-docs/?UYei3x) [Kindrachuk, J. *et al.* Ebola virus modulates transforming growth factor β signaling and cellular markers of mesenchyme-like transition in hepatocytes. *J. Virol.* **88**, 9877–9892 (2014).](https://www.zotero.org/google-docs/?UYei3x)

[73.](https://www.zotero.org/google-docs/?UYei3x) [Younan, P. *et al.* Ebola Virus Binding to Tim-1 on T Lymphocytes Induces a Cytokine Storm. *mBio* **8**, e00845-17 (2017).](https://www.zotero.org/google-docs/?UYei3x)

[74.](https://www.zotero.org/google-docs/?UYei3x) [Wiercińska-Drapalo, A., Flisiak, R., Jaroszewicz, J. & Prokopowicz, D. Increased plasma transforming growth factor-beta1 is associated with disease progression in HIV-1-infected patients. *Viral Immunol.* **17**, 109–113 (2004).](https://www.zotero.org/google-docs/?UYei3x)

[75.](https://www.zotero.org/google-docs/?UYei3x) [He, L. *et al.* Interleukin-27 is differentially associated with HIV viral load and CD4+ T cell counts in therapy-naïve HIV-mono-infected and HIV/HCV-co-infected Chinese. *PloS One* **9**, e96792 (2014).](https://www.zotero.org/google-docs/?UYei3x)

[76.](https://www.zotero.org/google-docs/?UYei3x) [Stylianou, E. *et al.* Raised serum levels of interleukin-18 is associated with disease progression and may contribute to virological treatment failure in HIV-1-infected patients. *Clin. Exp. Immunol.* **132**, 462–466 (2003).](https://www.zotero.org/google-docs/?UYei3x)

[77.](https://www.zotero.org/google-docs/?UYei3x) [Pallikkuth, S., Parmigiani, A. & Pahwa, S. The role of interleukin-21 in HIV infection. *Cytokine Growth Factor Rev.* **23**, 173–180 (2012).](https://www.zotero.org/google-docs/?UYei3x)

[78.](https://www.zotero.org/google-docs/?UYei3x) [Kekow, J. *et al.* Transforming growth factor-beta and suppression of humoral immune responses in HIV infection. *J. Clin. Invest.* **87**, 1010–1016 (1991).](https://www.zotero.org/google-docs/?UYei3x)

[79.](https://www.zotero.org/google-docs/?UYei3x) [Tian, R. R. *et al.* IFN-λ inhibits HIV-1 integration and post-transcriptional events in vitro, but there is only limited in vivo repression of viral production. *Antiviral Res.* **95**, 57–65 (2012).](https://www.zotero.org/google-docs/?UYei3x)

[80.](https://www.zotero.org/google-docs/?UYei3x) [Bermejo-Martin, J. F. *et al.* Th1 and Th17 hypercytokinemia as early host response signature in severe pandemic influenza. *Crit. Care* **13**, R201 (2009).](https://www.zotero.org/google-docs/?UYei3x)

[81.](https://www.zotero.org/google-docs/?UYei3x) [Li, N. *et al.* Influenza viral neuraminidase primes bacterial coinfection through TGF-β-mediated expression of host cell receptors. *Proc. Natl. Acad. Sci. U. S. A.* **112**, 238–243 (2015).](https://www.zotero.org/google-docs/?UYei3x)

[82.](https://www.zotero.org/google-docs/?UYei3x) [Albayrak, N. *et al.* Distinct Expression Patterns of Interleukin-22 Receptor 1 on Blood Hematopoietic Cells in SARS-CoV-2 Infection. *Front. Immunol.* **13**, 769839 (2022).](https://www.zotero.org/google-docs/?UYei3x)

[83.](https://www.zotero.org/google-docs/?UYei3x) [Furci, F. *et al.* IL-33 and the Cytokine Storm in COVID-19: From a Potential Immunological Relationship towards Precision Medicine. *Int. J. Mol. Sci.* **23**, 14532 (2022).](https://www.zotero.org/google-docs/?UYei3x)

[84.](https://www.zotero.org/google-docs/?UYei3x) [Smail, S. W., Babaei, E., Amin, K. & Abdulahad, W. H. Serum IL-23, IL-10, and TNF-α predict in-hospital mortality in COVID-19 patients. *Front. Immunol.* **14**, 1145840 (2023).](https://www.zotero.org/google-docs/?UYei3x)

[85.](https://www.zotero.org/google-docs/?UYei3x) [Zamani, B., Najafizadeh, M., Motedayyen, H. & Arefnezhad, R. Predicting roles of IL-27 and IL-32 in determining the severity and outcome of COVID-19. *Int. J. Immunopathol. Pharmacol.* **36**, 3946320221145827 (2022).](https://www.zotero.org/google-docs/?UYei3x)

[86.](https://www.zotero.org/google-docs/?UYei3x) [Olajide, O. A., Iwuanyanwu, V. U., Lepiarz-Raba, I. & Al-Hindawi, A. A. Induction of Exaggerated Cytokine Production in Human Peripheral Blood Mononuclear Cells by a Recombinant SARS-CoV-2 Spike Glycoprotein S1 and Its Inhibition by Dexamethasone. *Inflammation* **44**, 1865–1877 (2021).](https://www.zotero.org/google-docs/?UYei3x)

[87.](https://www.zotero.org/google-docs/?UYei3x) [Domingo-Gonzalez, R., Prince, O., Cooper, A. & Khader, S. A. Cytokines and Chemokines in Mycobacterium tuberculosis Infection. *Microbiol. Spectr.* **4**, 10.1128 (2016).](https://www.zotero.org/google-docs/?UYei3x)

[88.](https://www.zotero.org/google-docs/?UYei3x) [Chandrashekara, S., Anupama, K. R., Sambarey, A. & Chandra, N. High IL-6 and low IL-15 levels mark the presence of TB infection: A preliminary study. *Cytokine* **81**, 57–62 (2016).](https://www.zotero.org/google-docs/?UYei3x)

[89.](https://www.zotero.org/google-docs/?UYei3x) [Montaner, S. *et al.* The Role of Extracellular Vesicles in Modulating the Host Immune Response during Parasitic Infections. *Front. Immunol.* **5**, 433 (2014).](https://www.zotero.org/google-docs/?UYei3x)

[90.](https://www.zotero.org/google-docs/?UYei3x) [Johnston, C. J. C. *et al.* A structurally distinct TGF-β mimic from an intestinal helminth parasite potently induces regulatory T cells. *Nat. Commun.* **8**, 1741 (2017).](https://www.zotero.org/google-docs/?UYei3x)

[91.](https://www.zotero.org/google-docs/?UYei3x) [Vacca, F. & Le Gros, G. Tissue-specific immunity in helminth infections. *Mucosal Immunol.* **15**, 1212–1223 (2022).](https://www.zotero.org/google-docs/?UYei3x)

[92.](https://www.zotero.org/google-docs/?UYei3x) [Oyegue-Liabagui, S. L. *et al.* Cytokine response in asymptomatic and symptomatic Plasmodium falciparum infections in children in a rural area of south-eastern Gabon. *PloS One* **18**, e0280818 (2023).](https://www.zotero.org/google-docs/?UYei3x)

[93.](https://www.zotero.org/google-docs/?UYei3x) [Ing, R., Gros, P. & Stevenson, M. M. Interleukin-15 enhances innate and adaptive immune responses to blood-stage malaria infection in mice. *Infect. Immun.* **73**, 3172–3177 (2005).](https://www.zotero.org/google-docs/?UYei3x)

[94.](https://www.zotero.org/google-docs/?UYei3x) [Fievet, N. *et al.* Plasmodium falciparum induces a Th1/Th2 disequilibrium, favoring the Th1-type pathway, in the human placenta. *J. Infect. Dis.* **183**, 1530–1534 (2001).](https://www.zotero.org/google-docs/?UYei3x)

[95.](https://www.zotero.org/google-docs/?UYei3x) [Glineur, C., Leleu, I. & Pied, S. The IL-33/ST2 Pathway in Cerebral Malaria. *Int. J. Mol. Sci.* **23**, 13457 (2022).](https://www.zotero.org/google-docs/?UYei3x)

[96.](https://www.zotero.org/google-docs/?UYei3x) [Liao, M., Wang, C., Zhang, M. & Qiao, K. Insight on immune cells in rejection and infection postlung transplant. *Immun. Inflamm. Dis.* **11**, e868 (2023).](https://www.zotero.org/google-docs/?UYei3x)

[97.](https://www.zotero.org/google-docs/?UYei3x) [Nosik, M. *et al.* Dynamics of Plasmatic Levels of Pro- and Anti-Inflammatory Cytokines in HIV-Infected Individuals with M. tuberculosis Co-Infection. *Microorganisms* **9**, 2291 (2021).](https://www.zotero.org/google-docs/?UYei3x)

[98.](https://www.zotero.org/google-docs/?UYei3x) [Diedrich, C. R. & Flynn, J. L. HIV-1/Mycobacterium tuberculosis Coinfection Immunology: How Does HIV-1 Exacerbate Tuberculosis? *Infect. Immun.* **79**, 1407–1417 (2011).](https://www.zotero.org/google-docs/?UYei3x)

[99.](https://www.zotero.org/google-docs/?UYei3x) [Babu, S. & Nutman, T. B. Helminth-Tuberculosis Co-infection: An Immunologic Perspective. *Trends Immunol.* **37**, 597–607 (2016).](https://www.zotero.org/google-docs/?UYei3x)

[100.](https://www.zotero.org/google-docs/?UYei3x) [Mahajan, S., Mahajan, S. & Patgiri, S. Association and Interaction of Epstein–Barr Virus with SARS-CoV-2 Infection—A Review. *Viruses* **17**, 903 (2025).](https://www.zotero.org/google-docs/?UYei3x)

[101.](https://www.zotero.org/google-docs/?UYei3x) [Chen, T., Song, J., Liu, H., Zheng, H. & Chen, C. Positive Epstein–Barr virus detection in coronavirus disease 2019 (COVID-19) patients. *Sci. Rep.* **11**, 10902 (2021).](https://www.zotero.org/google-docs/?UYei3x)

[102.](https://www.zotero.org/google-docs/?UYei3x) [Hartgers, F. C. & Yazdanbakhsh, M. Co-infection of helminths and malaria: modulation of the immune responses to malaria. *Parasite Immunol.* **28**, 497–506 (2006).](https://www.zotero.org/google-docs/?UYei3x)
